# Exploring Patient, Parent and Clinician Views of Outcomes for Family-Centered Care in Neonatal Settings: A Qualitative Study

**DOI:** 10.3390/children13010156

**Published:** 2026-01-22

**Authors:** Cansel Kocakabak, Agnes van den Hoogen, Jos M. Latour, on behalf of the COUSIN Study Group

**Affiliations:** 1 School of Nursing and Midwifery, Faculty of Health, University of Plymouth, Plymouth PL4 8AA, UK; 2 Department Women and Baby, Neonatology, Wilhelmina Children’s Hospital, University Medical Centre Utrecht, 3584 EA Utrecht, The Netherlands; ahoogen@umcutrecht.nl; 3 Department of Nursing, Zhongshan Hospital, Fudan University, Shanghai 200032, China; 4 Curtin School of Nursing, Curtin University, Perth, WA 6102, Australia

**Keywords:** family-centered care, neonatology, outcomes, infants, parents, health personnel, focus group, framework analysis, qualitative research

## Abstract

**Highlights:**

**What are the main findings?**

- Qualitative analysis of family-centered care experiences, drawn from perspectives of parents, a former neonatal patient, and healthcare professionals, identified 42 distinct outcomes which were categorized into five domains.
- The five domains, with distinct outcomes, highlight the multidimensional family-centered care including: emotional functioning, role functioning, delivery of care, physiological health, and hospital resources.

**What are the implications of the main findings?**

- These findings show the importance of involving parents, former neonatal patients and healthcare professionals in the development of a robust core outcome set for family-centered care interventions in neonatal settings.
- The findings provide evidence for more consistent and meaningful evaluation of family-centered care research and practice in neonatology.

**Abstract:**

Background/Objectives: A neonatal intensive care units (NICU) admission of a premature infant is lifesaving; however, it can also be emotionally devastating experiences for parents. Family-centered care (FCC) interventions are designed to support parents and infants in the NICUs by integrating families into care delivery through partnerships with healthcare professionals. Heterogeneity in outcome reporting across FCC studies limits comparability. Developing a core outcome set (COS) for FCC is essential to address this gap. Aim: The aim of this study was to explore the views of former neonatal patients, parents, and healthcare professionals who have experiences with FCC in neonatal settings and elucidate outcomes that are important to them. Methods: This study followed the Core Outcome Measures Effectiveness Trial Handbook, which suggests involving stakeholders in identifying outcomes to reflect what is important to them rather than to researchers. Nine focus group discussions were conducted with 27 international key stakeholders from multiple countries (former neonatal patient *n* = 1; parents *n* = 8; healthcare professionals *n* = 18), reflecting FCC experiences across different neonatal settings. Data were analyzed using a modified framework analysis. Findings: Five outcome domains were identified including 42 distinct outcomes: (1) Emotional functioning/wellbeing of parents, infants, and healthcare professionals, reflecting emotional responses to a NICU admission of an infant; (2) Role functioning of parents, healthcare professionals, and others, highlighting that FCC strengthens their roles; (3) Delivery of care, highlighting the role of staff attitudes and organizational factors in supporting FCC; (4) Physiological health, reflecting infant physical health; (5) Hospital environment and resource use, reflecting healthcare utilization outcomes. Conclusions: Participants’ experiences provide meaningful insights into outcomes that should be evaluated in neonatal research and practice. These findings will inform the development of a COS for FCC in neonatal settings.

## 1. Introduction

The survival rates of infants have improved significantly with advances in medical technology and neonatal care [1]. As survival has increased [2], the number of infants admitted to neonatal intensive care units (NICUs) has also risen [3]. According to a joint report by UNICEF and the World Health Organization in 2020, an estimated 30 million newborns worldwide require NICUs care each year [4]. While NICU admission is lifesaving for infants [5], it can disrupt the development of the parent-infant relationship [6]. This experience can negatively affect both parents’ and infants’ psychological and physical health [7,8] while also encompassing broader relational and functional challenges during and following NICU admission [9,10]. Therefore, family-centered care interventions are essential to protect and promote the health and well-being of both parents and infants.

Family-centered care (FCC) is a philosophy [11] and an approach to healthcare that recognizes parents as integral members of the healthcare team [12]. FCC interventions in neonatal care vary widely across countries and include educational programs, family support, communication strategies, and environmental changes [13]. Despite variations, evidence shows that FCC interventions improve both parental mental health and infant physical and mental health outcomes [14]. However, a systematic review and meta-analysis by Ding et al., (2019) emphasized the inconsistency and heterogeneity of reported outcomes in FCC studies in NICUs [15]. This has led to limitations in comparability and synthesizing of outcomes across studies, which prevents the development of evidence-based guidelines. This is further challenged by interchangeable use of terms such as Family Integrated care (FICare), Creating Opportunities for Parent Empowerment (COPE), different content, and outcomes measures [15,16]. Thus, developing a standardized core outcome set (COS) is essential for FCC to ensure consistency in outcome measurement and to advance evidence-based FCC research and practices in neonatal settings.

A COS is a minimum set of outcomes that should be measured and reported in all trials for a specific health condition [17]. Developing a COS for FCC interventions in neonatal care will likely reduce heterogeneity of reported outcomes and facilitate evidence synthesis. This in turn can reduce research waste and enhance the translation of research findings into neonatal clinical practice through its contribution to guideline development and quality improvement projects. To ensure the relevance and uptake of a COS, inclusive stakeholder involvement is recommended, including patients and representatives from diverse healthcare systems and income settings [18]. The COUSIN (developing a core outcome set and outcome measures of FCC in neonatal care) project was therefore developed as an international initiative to address this gap.

For this project, we adhered to the Core Outcome Measures Effectiveness Trials (COMET) handbook guideline [19] and the Core Outcome Set Standards for Development (COS-STAD) [20] to ensure methodological rigor and transparency in the development of the COS. The initial step involves identifying outcomes to create a comprehensive list that will inform the consensus process. A quantitative systematic review has already identified outcomes reported in FCC studies [16]. However, COS-STAD also recommends involving stakeholders who have experience with or affected by the condition to propose outcomes that are important to these groups. Including former neonatal patients allows the identification of outcomes reflecting long-term impact of neonatal care. Therefore, the aim of this study was to explore the views of former neonatal patients, parents, and healthcare professionals with experience of FCC in neonatal settings and to elucidate outcomes that are important to them.

## 2. Materials and Methods

### 2.1. Study Design

This qualitative study used focus group discussions to explore the views of international stakeholders who have experience with FCC in neonatal care, as this approach is appropriate for eliciting diverse perspective and helps identify relevant outcomes [21]. We adopted a generic qualitative approach with an interpretive element to provide flexibility in capturing participants’ views and identifying meaningful outcomes without being restricted by a specific theoretical framework [22]. This approach was considered appropriate given that this study did not aim to generate theory or focus on lived experience in depth. The study was informed by a pragmatic orientation, acknowledging that knowledge is co-constructed through participants’ discussions. Data were collected through focus group discussions and analyzed using a modified framework analysis, consistent with its flexible and iterative nature described by Ward et al., (2013) [23], to support outcome identification and refinement. The Consolidated Criteria for Reporting Qualitative Research (COREQ) guideline was used to report this study [24], as it specifically designed to enhance transparency and rigor in reporting qualitative studies. Ethical approval was granted by the University of Plymouth Research Ethics Committee (reference number: 5385; ethical approval date 18 September 2024).

### 2.2. Study Setting

Nine focus group discussions were conducted online via Zoom between December 2024 and January 2025. This online format was chosen to facilitate flexibility for participants from diverse geographical and professional backgrounds and allowed the meetings to be scheduled at convenient times for participants in different time zones and world areas (Oceania-Asia; Europe-Africa; Northern and Southern America). All focus group discussions were conducted in English.

### 2.3. Participants

Participants were recruited internationally through the Global Foundation for the Care of Newborn Infants (GFCNI), a global organization dedicated to improving neonatal care and family health worldwide. An international sample was sought to capture diverse FCC experiences across different healthcare context. Participants consisted of two stakeholder groups: parents (including one former neonatal patient) and healthcare professionals. The parent group consisted of individuals who had experienced FCC during their infant’s hospital stay. One former neonatal patient was also included to provide a unique perspective reflecting longer-term outcomes of neonatal care and met the same selection criteria as other participants, no additional selection criteria were applied. Healthcare professional group had experienced delivering FCC in neonatal settings and were actively involved in implementing, supporting, or advocating for FCC as part of their professional practice. Experience with FCC was determined based on participants’ involvement within the GFCNI network rather than participation in a formal FCC program. All participants were required to be over 18 years of age and have sufficient proficiency in spoken English. Exclusion criteria were individuals under the age of 18 and those who were not able to communicate in English.

A total of 49 participants were invited to take part in the study, of whom 28 expressed interests, and 27 participated across the nine focus group discussions. Each focus group consisted of a minimum of two and a maximum of five participants.

### 2.4. Recruitment

We employed a convenience sampling technique, which was appropriate for recruiting participants through the GFCNI network. A parent representative from GFCNI distributed an email invitation including an online Joint Information Systems Committee (JISC) survey platform, which hosted the participant information sheet, consent form, and demographic information form. The demographic information form collected participants’ email address, and availability. After participants completed the JISC link and electronically signed consent, the researcher (CK) distributed a Doodle poll to schedule the focus group meetings at times convenient for participants.

### 2.5. Data Collection

The focus groups were conducted online using the Zoom platform, and the sessions lasted between 60–90 min. The discussions were moderated by a female PhD student (C.K.), who had training in qualitative research methods and had no prior personal or professional relationship with the participants. To support the moderator and take field notes, a second member of the research team (A.v.d.H. or J.M.L.) with experience in conducting focus group discussions attended the sessions as note taker.

The Zoom platform automatically generated audio and video recordings. Reflective notes were taken by the note taker during the focus group discussions and subsequently integrated into the analysis to support transparency and enhance the credibility of interpretation. Recordings were transcribed verbatim and pseudonymized using alphanumeric identification codes to protect participant confidentiality.

### 2.6. Data Analysis

A modified framework analysis approach was employed, consistent with its flexible and iterative nature described by Ward et al. (2013) [23], to support outcome identification and refinement. Following familiarization with the transcripts, the researcher (CK) conducted line-by-line coding in NVivo software (version 14) to support systematic outcome identification.

Through line-by-line coding, two types of data were identified: (1) experiential narratives describing the lived experiences and views related to FCC in neonatal care, and (2) explicit statements identifying outcomes they considered important. Experience-based narratives were coded inductively to generate initial outcomes, while participant-generated outcomes were extracted directly from transcripts.

All identified outcomes were initially mapped to relevant outcome domains in the COMET taxonomy (e.g., physiological, life impact and resource use). However, outcome domains were modified to better reflect the experiential and relational aspects of FCC within the neonatal context. These modifications were data-driven and informed by iterative discussion within research team. Inductively generated outcomes were compared, grouped, and iteratively refined through discussion to form coherent outcome domains that reflected shared patterns across stakeholder experiences. Two researchers (A.v.d.H. and J.M.L.) were involved throughout the analysis, including mapping of outcomes and refinement of outcome domains. Any discrepancies in coding or outcome domain allocation were discussed and resolved through consensus within research team. Discussions with the research team were held regularly to ensure rigor and credibility, and to support reflexivity during data interpretation.

The narratives in the Section 3 are presented with participant identifiers. The identifier FGD-1 reflects the first Focus Group Discussion (FGD). FGD-2 and so on, reflects the consecutive focus group discussions. PF is Participant Former neonatal patient; PD is Participant Doctor; PM is Participant Mother; PN is Participant Nurse.

### 2.7. Patient and Public Involvement

The wider core outcome set (COS) project involves GFCNI (Global Foundation for the Care of Newborn Infants), as our patient and public advisory group. A parent representative from GFCNI, who have knowledge of FCC and experience in parent advocacy, contributed to the development of the study protocol, documents such as consent form and participant information sheets, and advised on the refinement of the focus group discussion guide prior to data collection to ensure relevance and clarity.

## 3. Results

### 3.1. Participant Characteristics

A total of 27 individuals participated in nine focus group discussions: eight parents and one former neonatal patient, and 18 healthcare professionals (HCPs). All parent participants were mothers. Although recruitment targeted participants across all six continents, the final sample was predominantly based in Europe, with no participation from South America (Table 1).

### 3.2. Identification of Core Area, Outcome Domains and Outcomes

From the focus group discussions, we identified a total of 42 outcomes. Outcomes were generated through responses from two complementary sources: (1) outcomes derived through interpretive analysis of participants ‘experiential narratives describing their lived experiences of FCC in neonatal settings, and (2) participants’ explicit statements in response to questions which outcomes they considered important evaluating FCC. Outcomes were organized using the COMET taxonomy. The three core areas were adopted directly from the taxonomy, while outcome domains were reviewed and refined to ensure relevance of FCC. Subsequently, the identified outcomes were mapped to five outcomes domains across three core areas: Life Impact (three outcome domains), Physiological/Clinical (one outcome domain), and Resource Use (one outcome domain) (Table 2). All supporting participant quotations are provided in Electronic Supplemental Material Table S1.

#### 3.2.1. Outcome Domain: Emotional Functioning/Wellbeing of Parents, Infants, and Healthcare Professionals

This domain captures the emotional responses of parents, infants, and HCPs. Participants described premature birth and NICU admission as traumatic not only for parents and infants, but also for HCPs.

Participants reported that parents often experienced a persistent emotional burden from premature birth through to after discharge. They commonly reported emotional response, including shock, guilt, fear, loneliness, stress depression, and anxiety. Participants expressed that parent, particularly mothers, felt guilt related to premature birth and were shocked when confronted with the unfamiliar and highly technological NICU environment. They also described fear regarding their infant’s survival and possible deterioration, as well as loneliness arising from separation from their partner and other children. One mother expressed:

*‘As a parent, you’re completely quite lost. You’re in shock. You’re absolutely you know petrified and scared of this whole new environment and what’s happening to your baby. Then you’ve got all the guilt feelings and all of those things and trying to still function as a family if you have other children’.*

(PM001/FGD-2)

Participants further identified that parents experienced stress due to managing the care of their medically fragile infant at home and one nurses mentioned:

*‘The baby’s starting to be prepared for home parents are usually more stressed, show a lot of signs of anxiety’.*

(PN009/FGD-6)

Participants also highlighted that the emotional impact of the NICU extended to HCPs. They could experience stress, burnout, and fear while working, a one mother said:

*‘We cannot forget the needs of the providers, the needs of the ummm clinicians and professionals and realizing that just in my experience, they’ve also expressed that they’re fearful and overwhelmed’.*

(PM005/FGD-6)

Finally, FCC was described by the participants as a healing approach that could be as meaningful as medical treatment. They emphasized that FCC helps reduce negative emotional experiences and supports the emotional well-being of parents, infants, and healthcare professionals during neonatal care.

#### 3.2.2. Outcome Domain: Role Functioning of Parents, Healthcare Professionals, and Others

This outcome domain reflects the experience in the neonatal unit influencing the roles of parents and HCPs, and how FCC interventions shaped and supported these roles.

Participants described that parents should be actively involved in their infant’s care. They clarified that parental involvement did not refer to performing medical tasks but rather to participating in daily caregiving activities, such as changing diapers, expressing breast milk, and providing kangaroo care. Parental involvement was identified as an opportunity to strengthen the bonding between parents and infants, emphasizing involvement in their infant’s care as early as possible. The former neonatal patient explained experiencing delayed connection:

*‘When I speak to other adults who were born in the same kind of period. And we… have a feeling that you were… don’t really belong to your own family. So, it feels you have long for a long time you just felt like you were not really adopted but not really adopted you just were put in the family. And you don’t really belong to each other. How do you say you feel sometimes lonely in your own family and it’s uh that’s what the other adults around my own age have the same kind of feeling*’.

(PF001/FGD-2)

Participants emphasized that parents should be treated as primary caregivers rather than visitors. Parental involvement in their infant’s care strengthens their parental role. Participants highlighted that parents and infants should not be separated, and zero separation is ideal. They described that parents should be able to stay together with their infants as much as possible throughout the NICU stay:

*‘it’d be a perfect world, wouldn’t it though where there was no separation and yeah, they could all be together whilst they’re getting their intensive care*’.

(PM001/FGD-2)

One measurement of FCC success was described through increased opportunities for meaningful interaction between parents and infants. One nurse highlighted that parents could realize changes in their infants more readily:

*‘I feel that we succeed when we see the parents respond to the baby’s cues every time and we see that there is something that we see and we also knew that we succeeded when the parents brought late onset sepsis before we even see it in any tests. And that means that they know they’re a baby. and then they can yes read their cues read their signals and respond to them*’.

(PN007/FGD-4)

The crucial role of HCPs role in shaping parental experiences in NICUs was acknowledged by the participants, particularly through emotional, informational, and practical support. Key moments along the hospital journey for parents was requiring an increase emotional and practical support. One source of support was mentioned through peer networks, family members, and close friends:

*‘We need to let the parents decide who’s important for them to be with them at the NICU because we always say that the parents should be at the NICU but perhaps for the mother her mother or a friend is more important for her than the father at the time. So, I think we need to ask them and let another person than the parent visit the NICU’.*

(PN005/FGD-3)

These findings demonstrate that parents’ social networks play a crucial role in supporting their emotional well-being and helping them to cope with difficult situations.

#### 3.2.3. Outcome Domain: Delivery of Care

This outcome domain reflects how FCC should be provided to parents and delivered by HCPs. The attitudes of HCPs towards FCC were considered more important than room design, facilities and policies. Yet, narratives depicted considerable variation staff attitudes. Some staff did not perceive FCC as part of their professional duties and focused only on medical tasks, creating inconsistency in care and confusion among parents. Staff attitudes were further affected by misbeliefs such as losing professional roles, increased infection risk, and increased their workload, as reflected by a nurse:

*‘Some nurses, well, the parents … they steal my work. But well, your work is changing*’.

(PN005 FGD-3)

And a doctor expressed:

*‘There are some who are still resistant to that thinking ummm and they are more concerned about the infection control. when you have a lot of visitors or a lot of family members going in and out of the NICU. So, for them, that might introduce infection to the other patient’.*

(PD005/FGD-6)

Conversely, participants described staff resistance could decrease as they recognized the benefits of FCC. A head nurse said:

*‘As a head nurse, I think maybe the nurse the bad scenario will not like it. But now they are now like it because the parents come in and they can come forward to the babies. And the nurse think they have … umm their workload is their workload is lower’.*

(PN012/FGD-7)

Education and training were identified as key strategies for transforming staff attitudes of FCC. This included building competence in cultural and religious practice, as well as interpersonal skills. Participants reported that the communication style of staff shaped their ability to establish trusting relationships and meaningful interactions with parents. Empathic, supportive, non-judgmental, respectful, and reassuring communication style was perceived to foster parental trust and encourage their engagement in infant care staff. Also, the communication process, such as timing, clarity, and repetition, were considered to enhance parental understanding and supporting in their decision-making. Language barrier was identified as a communication challenge between staff and parents.

*‘it’s we become more families with refugee or migration background and then the communication gets really difficult because normally you need aaaaa a translator uh so we don’t have them in the ward 24 hours and so on’.*

(PD002/FGD-4)

Participants described that parents should be actively involved in care planning and decision-making, especially through sharing their observations, asking questions and participating in daily rounds. This level of participation promoted parental engagement and supported the development of collaborative relationships with healthcare professionals. They also highlighted differences in the level of involvement of parents and extended family members in decision-making across countries:

*‘Family-centered care means involving the parents in decision making or also in the care of the infants and also to a large extent from African perspective also involving the extend family’.*

(PN010/FGD-3)

Participants described that HCPs should teach parents practical caregiving skills and provide simple, clear explanations of rationale behind these practices. They highlighted that receiving adequate education and information during the NICU stay helped parents feel more prepared the discharge and the transition to home. In addition, other social and contextual factors for implementing FCC were highlighted, such as welcoming unit policies, space in the NICU, the presence of single rooms, family rooms, hospital facilities, visiting hours, cultural beliefs, transportation cost, restricted paternity leave, and having other children at home. These factors varied across countries and participants described their experiences from different countries such as one nurse from China said:

*‘They had 13 babies in a space of about 10 feet by 10 feet. And I just find there is so many babies. So, you can’t do family-centered care in that space’.*

(PN008/FGD-1)

Participants explained that travel distance, lack of accommodation opportunities, and facilities varied widely across countries and affected the parental involvement and presence. They further highlighted that although some hospital policy allows 24/7 access, parents cannot stay during the night because of staff reluctance.

#### 3.2.4. Outcome Domain: Physiological Health

This outcome domain covers the infant’s physical health. Overall, during the nine focus group discussions, the participants described several infant’s health related outcomes in their narratives. These specific outcomes were identified and noticed by the researchers while participants’ stories emerged on several topics related to other outcome domains. These physiological outcomes as mentioned in the participants’ narratives throughout the discussions are listed in Table 2.

Participants further discussed the infant short- and long-term outcomes. The short-term outcomes were associated with infant’s physical health and the clinical stability during the NICU stay, whereas long-term physical outcomes were considered as having less morbidity, as one mother said:

*‘the long term is the sequels because we know that reduced is different kinds of sequels*’,

(PM004/FGD-3)

and better psychological health in the infant’s future life another mother added:

*‘It’s a long-term outcome that it is very important also that has to do with social and umm I mean, the life of the babies as adults that they have been, they have been treated with family-centered care in comparison with the standard care babies that the differences in self-confidence, in success professional, success personal life, healthy personal life aaa things like that’.*

(PM002/FGD-4)

However, they highlighted that long term outcomes were influenced by many contributing factors beyond neonatal care, including growth and developmental changes, the emergence of secondary health conditions, family environment, and educational experiences.

#### 3.2.5. Outcome Domain: Hospital Environment and Resource Use

Participants perceived that FCC could contribute to reduced length of hospital stay, lower rates of readmission, and fewer emergency department visits, which collectively lead to cost savings and more efficient use of hospital resources. Some doctors described this as:

*‘length of stay is the number one immediate outcome because it’s the one you can use with hospital administrators to tell them we can save you money’.*

(PD001/FGD-1)

*‘hard to prove but I believe if we introduce family-centered care maybe cost for running NICU getting less’.*

(PD004/FGD-2)

They further suggested that these benefits could support the wider acceptance, standardization, and implementation of FCC in hospital settings, and national health policies.

## 4. Discussion

This study aimed to identify outcomes derived from FCC experiences of key stakeholders and determine which outcomes they consider important. We identified 42 outcomes and categorized them into three core areas and five outcome domains. These outcomes demonstrate that FCC is a holistic and interconnected approach that extends beyond infants’ clinical outcomes and include also parents, staff, and system-level factors. Importantly, the outcome domains provide insights into perceived impacts of an infant’s NICUs admission on parents-infants relationship, including bonding parents’ involvement in infant care, as well as the conditions required for FCC to be effectively implemented. In addition, our results underscore the importance of involving key stakeholders in the development of a robust core outcome set for FCC in neonatal care.

Although FCC is conceptually considered to include multiple aspects, such as education, support, and environmental factors, studies evaluating FCC have focused predominantly on infant and parent outcomes. Recently, a mixed-method systematic review similarly reported only infant clinical and parent psychosocial outcomes, with limited attention to staff and health system level outcomes [25]. Evidence on how FCC influences staff and organizational level outcomes remain comparatively underexplored in trials. Such outcomes may include staff burnout, staff turnover, team communication, and unit culture, all of which are essential for effective FCC implementation but are rarely captured in trials. Our study contributes to addressing this gap by identifying outcomes across all levels and highlighting the need for FCC to adapt a multi-level relational perspective that reflects the complexity of real-world neonatal care.

The domain emotional functioning/wellbeing of parents, infants and staff comprise outcomes relevant to the emotional challenges experienced by all stakeholders. Consistent with a previous study exploring parents’ experiences, our parent-related outcomes indicate that parents may experience different emotional responses at each phase, from the NICU to home and these responses can reveal in a range of distinct outcomes [26,27]. Similarly, infant-related outcomes align with existing evidence showing that NICU admission and medical procedures can be a source of distress for infants [28]. Our staff-related outcomes depict that staff can further experience emotional strain. Staff emotional-well-being is important for ensuring the quality and continuity of FCC implementation. Therefore, our findings highlight the need for these outcomes to be explicitly incorporated into an outcome list to capture the experiences of key stakeholders involved in neonatal care.

Our role-related outcomes are consistent with existing literature showing that FCC develops parental role by strengthening parent-infant bonding, improving parental confidence in caregiving, and enhancing parents’ ability to cope with emotionally challenging situations [29]. Parental presence emphasizes that parents should stay with their infants regardless of age or stability [30]. Parental presence and involvement such as breastfeeding, touching, and singing reduces infant stress [31], and improves neurodevelopment [32], and physiological stability [33]. The role of staff in coaching, guiding, and supporting [34,35] as parents gain skills and confidence for their infants’ care is consistent with prior FCC evidence, including the use of diaries for parents [36]. Furthermore, outcomes related to support from peers, family, and friends align with earlier studies [37,38] highlighting the importance of social networks for parents in reducing stress and enhancing caregiving. Our findings demonstrate that the role-related outcomes within FCC are based on reciprocal relationships, strengthen those relationships, and supported within broader family and social systems. These outcomes identified relevant to role-functioning in our study are consistent with established FCC evidence and support the relevance outcomes for inclusion in FCC evaluation [15].

The delivery of care outcome domain highlights that effective FCC implementation depends on staff attitudes, communication, education, shared-decision-making, and organizational factors [39]. Consistent with previous studies, staff attitudes in our findings can act either as facilitators [40] that enhance the quality of FCC or as barriers [41] when parental involvement is perceived, as a threat to professional roles [42] or as an additional workload [43]. Staff education and training are required for changing staff mindset to deliver FCC based on the needs and wishes of infants and parents [44]. However, educating staff only is not sufficient to deliver the quality of FCC, parents also require education about their infants’ care and treatment [45] and how to communicate effectively with staff [46]. Communication plays an important role in shaping a trusting relationship between parents and staff [47] and in involving parents in decision-making about their infant’s care [48]. Additionally, organizational factors in our findings are consistent with previous studies, such as physical layout, welcoming unity, and staff levels [49,50]. These findings identify what is needed to support effective FCC, and their inclusion in the COS is essential to ensure the delivery of high-quality of FCC.

### Strengths and Limitations

A strength of our study is the inclusion of a diverse group of stakeholders with international backgrounds cultures and experiences. A further strength is the inclusion of a former neonatal patient, which added a unique long-term lived experience perspective alongside parents and healthcare professionals. Although some focus groups consisted exclusively of healthcare professionals or parents, other discussions included both groups. This overall design enabled the integration of multiple perspectives, providing a comprehensive understanding of FCC outcomes. Convenience sampling supported the recruitment of participants, and reduced power imbalances among stakeholders. The use of a modified framework analysis combined with the COMET taxonomy strengthened the rigor and systematic nature of outcome identification.

Some limitations must be acknowledged. Despite the international scope, the sample size was small and not globally representative. Most of participants were from Europe, with no participation from South America, limiting the geographic diversity of sample. Parental participants were exclusively mothers, which limits gender diversity within the parent group. In addition, the inclusion of only one former neonatal patient limits the extent to which this stakeholder perspective is represented. In qualitative research, the analysis is shaped by subjective interpretations. This limitation hinders generalizability of the findings.

## 5. Conclusions

We identified 42 outcomes across five domains that stakeholders considered essential for evaluating FCC, underscoring its complex and multidimensional nature. These findings will directly inform the development of a comprehensive COS and promote more inclusive and meaningful outcome assessment in neonatal research. This study emphasized that stakeholder-driven outcomes can provide a foundation for advancing FCC research and improving the quality of FCC in clinical practice. Further research should focus on consensus building, involving key stakeholders in an e-Delphi survey to refine and prioritize outcomes, and online consensus meeting to finalize a core outcome set for FCC research in neonatal settings.

## Figures and Tables

**Table 1:** Participants demographic data.

Characteristics	Parents *n* = 8	Former Neonatal Patient *n* = 1	Healthcare Professionals *n* = 18
**Sex**			
Male	0		7
Female	8	1	11
**Age**			
26–35	0		3
36–45	1	1	3
46–55	4	0	3
56–65	1	0	5
66–75	1	0	4
Not reported	1	0	0
**Race/Ethnicity**			
White	6	1	12
Black	2	0	1
Asian	0	0	5
**Geographical region**			
Asia	0	0	5
Europe	6	1	10
Africa	1	0	1
North America	0	0	2
Oceania	1	0	0
**Professional role**			
Doctor	-	-	6
Nurse	-	-	12
Physiotherapist	-	-	1

**Table 2:** Core areas, outcome domains, and outcomes mapped using the COMET taxonomy.

Core Area	Outcome Domain	Outcomes
Life Impact	Emotional functioning/wellbeing of parents, infants and healthcare professionals	Bonding, Parental stress, Parental anxiety, Depression, Staff burnout, Staff stress, Parental fear, Parental guilt, Infant stress, Infant discomfort
	Role functioning of parents, healthcare professionals, and others	Parental involvement in infant care, Parental role, Parental presence, Parental preparedness discharge, Parental confidence, Parental self-efficacy, Staff coaching, Peer support, Family support, Friend support
	Delivery of care	Staff attitudes towards FCC, Staff workload, Infection, Staff education and training FCC, Staff communication, Shared decision making, Parental satisfaction, Parental trust, Parental advocacy Parental empowerment, Parental knowledge in their infant’s care and treatment
Physiological/Clinical	Physiological Health	Mortality, Infant pain, infant neurodevelopment, Necrotizing enterocolitis, Retinopathy of prematurity, Breastfeeding, exclusively breastfeeding at discharge, Weight gain, Growth
Resource Use	Hospital environment and resource use	Length of NICU stay, Readmission, Emergency department visit

## Data Availability

The raw data supporting the conclusions of this article will be made available by the authors on request.

## Supplementary Materials

The following supporting information can be downloaded at: https://www.mdpi.com/article/10.3390/children13010156/s1, Table S1: Core areas, outcome domains, illustrative quote from focus group discussions.

## Abbreviations

The following abbreviations are used in this manuscript:

FCC	Family-Centered Care
NICU	Neonatal Intensive Care Units
COS	Core Outcome Set
GFCNI	Global Foundation for the Care of Newborn Infants
COMET	Core Outcome Measures Effectiveness Trials
COS-STAD	Core Outcome Set Standards for Development
COREQ	Consolidated Criteria for Reporting Qualitative Research
NVivo	Qualitative Data Analysis Software

## References

[1] Çeri A., Gültekin N.D., Keskin D.M. (2025). Neonatal care in the twenty-first century: Innovations and challenges. World J. Pediatr..

[2] Sharrow D., Hug L., You D., Alkema L., Black R., Cousens S., Croft T., Gaigbe-Togbe V., Gerland P., Guillot M. (2022). Global, regional, and national trends in under-5 mortality between 1990 and 2019 with scenario-based projections until 2030: A systematic analysis by the UN Inter-agency Group for Child Mortality Estimation. Lancet Glob. Health.

[3] Harrison W., Goodman D. (2015). Epidemiologic Trends in Neonatal Intensive Care, 2007–2012. JAMA Pediatr..

[4] World Health Organization Nearly 30 Million Sick and Premature Newborns in Dire Need of Treatment Every Year. https://www.who.int/news/item/13-12-2018-nearly-30-million-sick-and-premature-newborns-in-dire-need-of-treatment-every-year.

[5] Sarathy L., Roumiantsev S., Lerou P.H. (2023). Who Needs the NICU? Trends and Opportunities for Improvement. Hosp. Pediatr..

[6] Dressler C. (2024). The psychological implications of having an infant in the NICU for mothers and fathers. J. Neonatal Nurs..

[7] Treherne S.C., Feeley N., Charbonneau L., Axelin A. (2017). Parents’ Perspectives of Closeness and Separation with Their Preterm Infants in the NICU. J. Obstet. Gynecol. Neonatal Nurs..

[8] Shaw R.J., Givrad S., Poe C., Loi E.C., Hoge M.K., Scala M. (2023). Neurodevelopmental, Mental Health, and Parenting Issues in Preterm Infants. Children.

[9] Greene M.M., Rossman B., Patra K., Kratovil A., Khan S., Meier P.P. (2015). Maternal psychological distress and visitation to the neonatal intensive care unit. Acta Paediatr..

[10] Lean R.E., Rogers C.E., Paul R.A., Gerstein E.D. (2018). NICU Hospitalization: Long-Term Implications on Parenting and Child Behaviors. Curr. Treat. Options Pediatr..

[11] Malusky S.K. (2005). A concept analysis of family-centered care in the NICU. Neonatal Netw..

[12] Waddington C., van Veenendaal N.R., O’Brien K., Patel N. (2021). Family integrated care: Supporting parents as primary caregivers in the neonatal intensive care unit. Pediatr. Investig..

[13] Lazzerini M., Bua J., Vuillard C.L.J., Squillaci D., Tumminelli C., Panunzi S., Girardelli M., Mariani I. (2024). Characteristics of intervention studies on family-centred care in neonatal intensive care units: A scoping review of randomised controlled trials. BMJ Paediatr. Open.

[14] O’Brien K., Robson K., Bracht M., Cruz M., Lui K., Alvaro R., da Silva O., Monterrosa L., Narvey M., Ng E. (2018). Effectiveness of Family Integrated Care in neonatal intensive care units on infant and parent outcomes: A multicentre, multinational, cluster-randomised controlled trial. Lancet Child. Adolesc. Health.

[15] Ding X., Zhu L., Zhang R., Wang L., Wang T.T., Latour J.M. (2019). Effects of family-centred care interventions on preterm infants and parents in neonatal intensive care units: A systematic review and meta-analysis of randomised controlled trials. Aust. Crit. Care.

[16] Kocakabak C., van den Hoogen A., Rothfus M., Campbell-Yeo M., Kostenzer J., Axelin A., Schofield P., Latour J.M. (2025). Identifying outcomes and outcome measures in neonatal family-centered care trials: A systematic review. Pediatr. Res..

[17] Williamson P.R., Altman D.G., Blazeby J.M., Clarke M., Devane D., Gargon E., Tugwell P. (2012). Developing core outcome sets for clinical trials: Issues to consider. Trials.

[18] Karumbi J., Gorst S., Gathara D., Young B., Williamson P. (2023). Awareness and experiences on core outcome set development and use amongst stakeholders from low- and middle- income countries: An online survey. PLoS Glob. Public Health.

[19] Williamson P.R., Altman D.G., Bagley H., Barnes K.L., Blazeby J.M., Brookes S.T., Clarke M., Gargon E., Gorst S., Harman N. (2017). The COMET Handbook: Version 1.0. Trials.

[20] Kirkham J.J., Davis K., Altman D.G., Blazeby J.M., Clarke M., Tunis S., Williamson P.R. (2017). Core Outcome Set-STAndards for Development: The COS-STAD recommendations. PLoS Med..

[21] Keeley T., Williamson P., Callery P., Jones L.L., Mathers J., Jones J., Young B., Calvert M. (2016). The use of qualitative methods to inform Delphi surveys in core outcome set development. Trials.

[22] Kahlke R.M. (2014). Generic Qualitative Approaches: Pitfalls and Benefits of Methodological Mixology. Int. J. Qual. Methods.

[23] Ward D.J., Furber C., Tierney S., Swallow V. (2013). Using Framework Analysis in nursing research: A worked example. J. Adv. Nurs..

[24] Tong A., Sainsbury P., Craig J. (2007). Consolidated criteria for reporting qualitative research (COREQ): A 32-item checklist for interviews and focus groups. Int. J. Qual. Health Care.

[25] Hodgson C.R., Mehra R., Franck L.S. (2025). Infant and Family Outcomes and Experiences Related to Family-Centered Care Interventions in the NICU: A Systematic Review. Children.

[26] Hua W., Zhou J., Wang L., Li C., Zheng Q., Yuwen W., Jiang L. (2023). ‘It turned my life upside down’: Parents’ emotional experience of the transition with their preterm infant from birth to discharge Home-A qualitative study. Aust. Crit. Care.

[27] van den Hoogen A., Eijsermans R., Ockhuijsen H.D.L., Jenken F., Oude Maatman S.M., Jongmans M.J., Verhage L., van der Net J., Latour J.M. (2021). Parents’ experiences of VOICE: A novel support programme in the NICU. Nurs. Crit. Care.

[28] Givrad S., Dowtin L.L., Scala M., Hall S.L. (2021). Recognizing and mitigating infant distress in Neonatal Intensive Care Unit (NICU). J. Neonatal Nurs..

[29] Cheng C., Franck L.S., Ye X.Y., Hutchinson S.A., Lee S.K., O’Brien K. (2021). Evaluating the effect of Family Integrated Care on maternal stress and anxiety in neonatal intensive care units. J. Reprod. Infant. Psychol..

[30] Roué J.M., Kuhn P., Lopez Maestro M., Maastrup R.A., Mitanchez D., Westrup B., Sizun J. (2017). Eight principles for patient-centred and family-centred care for newborns in the neonatal intensive care unit. Arch. Dis. Child. Fetal Neonatal Ed..

[31] Pillai Riddell R.R., Bucsea O., Shiff I., Chow C., Gennis H.G., Badovinac S., DiLorenzo-Klas M., Racine N.M., Ahola Kohut S., Lisi D. (2023). Non-pharmacological management of infant and young child procedural pain. Cochrane Database Syst. Rev..

[32] Synnes A.R., Petrie J., Grunau R.E., Church P., Kelly E., Moddemann D., Ye X., Lee S.K., O’Brien K. (2022). Family integrated care: Very preterm neurodevelopmental outcomes at 18 months. Arch. Dis. Child. Fetal Neonatal Ed..

[33] Pillai A., Pournami F., Prabhakar J., Nair P., Jain N. (2022). Effect of Early Parent Participation Program on Physiological Stability in Preterm Infants: A Randomized Controlled Trial. Am. J. Perinatol..

[34] Abukari A.S., Acheampong A.K., Aziato L. (2022). Experiences and contextual practices of family-centered care in Ghanaian nicus: A qualitative study of families and clinicians. BMC Health Serv. Res..

[35] Broom M., Parsons G., Carlisle H., Kecskes Z., Thibeau S. (2017). Exploring Parental and Staff Perceptions of the Family-Integrated Care Model: A Qualitative Focus Group Study. Adv. Neonatal Care.

[36] Muhsen W., Guillot Lozano A., Latour J.M. (2025). A Closer Look at Parental Narratives: A Qualitative Analysis of Parental Entries in Neonatal Research Diaries of Preterm Infants Participating in the REPORT-BPD Feasibility Study. Children.

[37] Pollock M.D., Ming D., Chung R.J., Maslow G. (2022). Parent-to-parent peer support for children and youth with special health care needs: Preliminary evaluation of a family partner program in a healthcare system. J. Pediatr. Nurs..

[38] Pereira N., MacDonald C., Drobot A., Bennett A., Ali A.-B., Garros D. (2021). A Peer and Volunteer Program for Patients and Their Families in the Pediatric Intensive Care Unit: A Pilot Program Evaluation. Front. Pediatr..

[39] Dall’Oglio I., Mascolo R., Portanova A., Ragni A., Amadio P., Fiori M., Tofani M., Gawronski O., Piga S., Rocco G. (2022). Staff Perceptions of Family-Centered Care in Italian Neonatal Intensive Care Units: A Multicenter Cross-Sectional Study. Children.

[40] Oude Maatman S.M., Bohlin K., Lilliesköld S., Garberg H.T., Uitewaal-Poslawky I., Kars M.C., van den Hoogen A. (2020). Factors Influencing Implementation of Family-Centered Care in a Neonatal Intensive Care Unit. Front. Pediatr..

[41] Abukari A.S., Schmollgruber S. (2024). Perceived barriers of family-centred care in neonatal intensive care units: A qualitative study. Nurs. Crit. Care.

[42] Benzies K.M., Shah V., Aziz K., Lodha A., Misfeldt R. (2019). The health care system is making ‘too much noise’ to provide family-centred care in neonatal intensive care units: Perspectives of health care providers and hospital administrators. Intensive Crit. Care Nurs..

[43] Vetcho S., Ullman A.J., Petsky H., Wiroonpanich W., Cooke M. (2023). Parent and interdisciplinary professional perceptions of family-centered care in Thai NICU: A qualitative study. Nurs. Crit. Care.

[44] Latour J.M., Hazelzet J.A., Duivenvoorden H.J., van Goudoever J.B. (2010). Perceptions of parents, nurses, and physicians on neonatal intensive care practices. J. Pediatr..

[45] Moreno-Sanz B., Alferink M.T., O’Brien K., Franck L.S. (2025). Family integrated care: State of art and future perspectives. Acta Paediatr..

[46] Benzies K.M. (2016). Relational Communications Strategies to Support Family-Centered Neonatal Intensive Care. J. Perinat. Neonatal Nurs..

[47] Hassankhani H., Negarandeh R., Abbaszadeh M., Craig J.W., Jabraeili M. (2020). Mutual trust in infant care: The nurses and mothers experiences. Scand. J. Caring Sci..

[48] Lorié E.S., Wreesmann W.W., van Veenendaal N.R., van Kempen A., Labrie N.H.M. (2021). Parents’ needs and perceived gaps in communication with healthcare professionals in the neonatal (intensive) care unit: A qualitative interview study. Patient Educ. Couns..

[49] McDonald R., Moloney W. (2023). Improving the Implementation of Family-Centered Care Within the Neonatal Care Unit: Empowering Parents to Participate in Infant Care. J. Perinat. Neonatal Nurs..

[50] Zanoni P., Scime N.V., Benzies K., McNeil D.A., Mrklas K. (2021). Facilitators and barriers to implementation of Alberta family integrated care (FICare) in level II neonatal intensive care units: A qualitative process evaluation substudy of a multicentre cluster-randomised controlled trial using the consolidated framework for implementation research. BMJ Open.

