# Supplementary material for: Exploring Patient, Parent and Clinician Views of Outcomes for Family-Centered Care in Neonatal Settings: A Qualitative Study
Source: Children (Basel). 2026 Jan 22;13(1):156. doi: 10.3390/children13010156 (PMC12839950; doi:10.3390/children13010156)
Supplement: Supplementary file 1 [file children-13-00156-s001.zip › children-4048620-supplementary.pdf]

Electronic Supplemental Material Table S1: Core Areas, Outcome domains, Illustrative Quotes from Focus Group Discussions

Core Area	Outcome Domain	Outcome	Illustrative Quotes	File	Participant Code
Life Impact	Delivery of care	Parental involvement restricted due to limited NICU space	The environment didn't make a difference. But again, we have the luxury. I just came back from China. And they had 13 babies in a space of about 10 feet by 10 feet. And I just find there is so many babies. So, you can't do family-centred care in that space. But we can, and it didn't matter for us.	FGD-1	PN008
		Parental involvement is linked welcoming unit policy	The second one is policy, the policies of the units with respect to our parents' welcome. Are they allowed to do certain things or are they restricted? Are they treated as visitors or are they treated as part of the team? All of those things are written in policies and unwritten in the culture of the NICU.	FGD-1	PD001
		Parental presence is linked maternity leave,<br>proximity to hospital,<br>transport access family support	There are societies where mothers can be with their babies all the time because they have family maternity leave. Umm Because they are nearby the hospital and there is public transportation, and they can get there umm because there are other people who can take care of their siblings so that they have time to come see the baby	FGD-1	PD001
		Family's psychosocial condition	the last one is there are individual issues you can build the best NICU, you can operate it in the best way possible and if the mom is a heroin addict. Umm You probably are going to have difficulty accomplishing you sometimes can't establish family-centred care.	FGD-1	PD001
	Role functioning	Peer support in open ward<br>Parental presence<br>Parent-infant interaction	The warmest memory I have of family-centred care is when I visited a NICU in Madrid, which had been built in the 1980. But in this NICU, which had big open rooms with 7 or 8 babies in each room. All the mothers were there. They were sitting around in a circle rocking their babies. It was the most nurturing environment for both babies and the mothers. I have ever seen. And it was done with the oldest physical design and facility that I had been in. So, absolutely, the physical environment is not an essential in order to accomplish it. However, I am	FGD-1	PD001

			absolutely sure that there are some families who will spend more time with their babies. If they have a private room, more willing to sing to them, to talk to them, to do skin to skin with them. When they have that privacy ownership. Umm It is their space; it is their family. Umm I am ..There is no doubt in my mind that there are families for whom that is optimal. And it is not all of them for sure. There are situations where the families never show up and that kid doesn't belong in a private room because then it is an isolation chamber.		
	Delivery of care	Staff attitudes towards FCC	And I had tried to have an interaction with nurse there and she just said, no, I can't talk to you. I won't talk to you. And so, to me, that is the biggest challenge not the space not the managers, not the families themselves, not the equipment. It is umm my nursing colleagues who resist, and I heard you know nurses, say, no, I just can't where the families are in the way. Get them out of here. You know in a NICU. And so, to me over the last 10, 12 years, that has been my biggest challenge.	FGD-1	PN008
		Staff attitudes toward FCC	if not the biggest there are nurses, again, older nurses in particular, but I don't think unique to them who really just want to give the baby its feeding or draw the lab work and then get back to checking their TikTok. umm There are other nurses who are very nurturing who find a lot of gratification from getting families involved and and seeing the baby go home to a good family and stay connected with that family. For years after the kid goes home if all our nurses could be that kind of nurse. I don't think we would be talking about this issue, but they aren't,. I don't see any likelihood that they will be in the future. So, what we must do is get to a point where this part of the care is **not considered optional.** Right now. We tell a nurse, you have to take the vital signs, you have to give the feeding, you have to draw this lab work at this particular time. That's what all the orders are. There is no order or expectation that you will be wonderful to families. you will encourage them every way you can. There's only the expectation that you won't be awful to families. And until that expectation changes, I think we will always be struggling with that issue. And it's not just nurses. Physicians are equally guilty, there are some who are wonderful with families and there are some for whom Talking to a family is the least pleasant part of their day and	FGD-1	PD001

			they... are clearly treated that way and do it as quickly as possible and move on...		
	Role functioning	Parental role<br>Parental involvement in their infant's care	And then the final component is the parents themselves, of course. And hmm You can lead a horse to water, but If they're not interested in being...the primary caregiver for their baby and and are quite happy to let somebody else be their baby's caregiver. then... we don't have much of a way to deal with that. So we can do the best to make it possible for them but Sometimes parents are still not going to be involved.	FGD-1	PD001
		Peer support	they have one whole nurse In China, they have one whole nurse who's dedicated just to family integrated care, but she does a lot of data collection. Aa for them. we don't have many paid positions in Canada. But what we do have in that psychosocial support aa component of family integrated care is something we call peer family mentors. And those are volunteered positions ummm Families. We're usually a year out after the birth of their birth aaa Typically preterm. Umm Though families of children with other kinds of aa umm challenges, conditions want to talk to a family who has a baby like theirs and we're having some difficulty with those kinds of matches. But the peer family mentors are volunteers. what it needs is what it requires is some training and coordination umm those peer family mentors volunteers serve in that role. Because I'm like. I am like the US, Canada has a publicly funded healthcare system, which is not financially in a very good situation.	FGD-1	PN008
		Parental role	Umm The idea of families though umm I don't know if you have as many newcomers in the US as we do in Canada. but from the newcomers from other countries. Where there's a lot of **hierarchy** in terms of you know healthcare systems, education systems the newcomer families see the doctors and the nurses as much more expert than they are and they just abdicate that role, that parenting role in the NICU because they don't see a place for themselves. So that's been hard work to get	FGD-1	PN008

			those families aaa to believe that they have a role in the NICU. But it's the newcomers' families who come from a very hierarchical system. But once they're in there, they're in there.		
	Delivery of care	Parent-staff communication	Because they've got all these translation machines. And anyone who actually speaks the dialect goes. That's not right. That's not what we were trying to tell you. So the perennials, they're just like, they're just really having a lot of trouble communicating. Aaa You know, the baby's progress with the families, with the newcomers. We call them newcomers.	FGD-1	PN008
		Staff cultural competence	but it's the lack of understanding of the cultural and religious traditions that go with caring for a newborn. that vex the nurses.	FGD-1	PN008
		Shared decision making	When we sit around deciding what management should be for the kid. Umm To the extent that families can provide some insight and go back to nutrition again. If we're deciding what to feed a baby, we want to know what the mother's choices with respect to breastfeeding and not only that but with respect to When she takes the baby home, does she plan to exclusively breastfeed? Is she going to give the kids some bottle of the pump breast milk? Is dad going to give that in the middle of the night, so mom can sleep. Is the baby going to a babysitter and they're going to be giving formula. The family has a big… role in deciding what we're going to do, certainly in the kids close to discharge. And in terms of all the other care decisions. Some of them there's no role for the family to help us decide what dose of antibiotic to use, for example, but to the extent that families can and should have essay in the baby's medical decisions. Family-centred care allows them, identifies them as an essential member of the decision making and then in delivering the care itself	FGD-1	PD001

	Role functioning	Parental involvement	it is the same aspect there are some elements of the care that families and wouldn't want to provide, such as starting an iv or intubating a baby. But there are other elements such as helping with feedings, calming the baby while an IV is being started or while a lab is being drawn. There are pieces of care where the family can be very helpful. And it's up to them whether they wish to be, but our responsibility would be to offer them to be part of that care team. And every piece of it that is possible. And then the part about holding their baby talking to their baby singing to their baby nurturing their baby. They are actually the primary source of that and we're just the backup.	FGD-1	PD001
Resource Use	Hospital resource and environment	LOS<br>Readmission<br>ED visit	But so it depends on who you're talking to. And I'm continually surprised when I walk into a room And I think, okay, I'm in a room with a lot of government folks here I'm going to talk about length of stay readmissions and ED visits, emergency department visits.	FGD-1	PN008
		Healthcare cost savings	We measured for the health system; we measure length of stay. We measured ED visits and we measured readmits Because we reduce length of stay by two and a half days in the trial. And that was sustained. We're still cutting it by about 7 8 days that avoided Oh, millions and millions of dollars, depending on how you calculate the per diem. The length of stay was their primary outcome. But the government constituents were interested in making sure we didn't download that early discharge to other parts of the health system. i.e. emerge and readmissions. And we didn't. In fact, at some point, we significantly reduced those with family integrated care under the umbrella of family-centred care	FGD-1	PN008
Life Impact	Emotional functioning/wellbeing	Parents mental health<br>Parental confidence	And they go, well, we really care about our constituents. And if they're satisfied with the care and If we've improved mental health and then going Okay, well, off we go. And now we're looking at the maternal mental health and paternal psychosocial distress and confidence.	FGD-1	PN008
		Parental psychosocial distress	Aaa For aaa for the mothers and the fathers, we have qualitative data for the fathers aaa and psychosocial distress is universal for both parents.	FGD-1	PN008

		Anxiety<br>Depression<br>Stress	aaa The literature says that We know that we measured aaa anxiety, depression, and stress is what we measured, and we measured. We're really coming to consensus on what those measures should be for parents in the NICU	FGD-1	PN008
		Parental knowledge and confidence<br>Self-efficacy	And We also measured umm knowledge and confidence or self-efficacy. umm I would choose a different No, what was that? No, I think we chose the right self-efficacy measure for this study.	FGD-1	PN008
	Delivery of care	Unit culture	What we didn't measure, which I think I would want to measure. if I were doing this again an outcome would be the outcome would be the unit culture And California has a really good measure of unit culture hierarchy specifically that captures this idea of how well the team works together.	FGD-1	PN008
		Relational communication	The other thing that I couldn't measure. That is an important outcome. I think for family integrated care, the way we deliver it is we teach relational communications. And I have seen anecdotally that… the relational communications that we teach the providers to use with the parents aaa aaaa has a trickledown effect. And the nurses among themselves and within the team are asking better questions. There are less lateral emotional attacks and more questioning	FGD-1	PN008
Life Impact	Emotional functioning/wellbeing	Parental burnout<br>Parental stress<br>Parental sleep<br>Reduction stress leave	I think I would, if I could do a real true family-centred intervention, I would want to measure if I could cut burnout or stress sleeps is what I would have been another outcome that I think. And if you want to show a health system that they've saved money if you could show a reduction in stress leave That's huge.	FGD-1	PN008
		Staff burnout<br>Staff stress	I just wanted to pop in because I am making notes throughout your burnout and stress is that measured among staff? Or is it among parents? PN008= We only measured it with parents. And I wish I'd have measured it with staff. We have qualitative data from the staff but It was pre-COVID. And I think we have different results today	FGD-1	PN008

	Delivery of care	Staff satisfaction with FCC<br>Staff turnover	They were misguided in their protection of their staff. If they really wanted to know this was a good idea for their staff. They would have been willing to ask them because I think the gratification that nurses get when family-centred care really works right is unmatched anywhere else in the hospital. And we have a lot of nurses that have been here for 20 or 30 years. Our turnover is much less. We have a waiting list for nurses waiting to come work at our unit. That's a problem that lots of other units wish they had. So, if nursing administration was really interested, umm they should have asked the nurses too how they felt about it.	FGD-1	PD001
Resource Use	Hospital environment and resource use	LOS<br>Cost savings	so length of stay is the number one immediate outcome because it's the one you can use with hospital administrators to tell them we can save you money. if you just invest at the front end in single family rooms, parents support people etc. The United States is terrible in long-term outcomes. Europe is much better. And those are the ones that really matter, of course. So how the baby's doing five years 10 years on, how the family is doing umm At that point, those outcome measures are the ones that the people sitting in the legislators legislatures can look at and say. We can save everybody money by investing at the front end. So those are the data that affect the decision makers at the hospital level short term at the national level long term that would actually allow family-centred care to become the standard.	FGD-1	PD001
Physiological/ Clinical	Physiological Health	Total parental nutrition<br>Full enteral feeding	You know, the decreased time on TPN, shorter time to full feeds.	FGD-1	PN008
Life Impact	Role functioning	Skin-to-skin contact	a shorter time to skin to skin, first skin to skin care And I can't even remember. I don't think I can even remember all the different outcomes that were significant	FGD-1	PN008
	Physiological Health	Apnoea	We didn't have a difference on the frequency of apnoea aaa	FGD-1	PN008

Physiological/ Clinical		Infection	One of the things that if I were going to pick new outcomes. I would look toward what are issues right now. And so infection is an issue. And we have data on... on the regional versus urban hospitals in terms of antibiotic use because in<br>our province, the regional hospitals are served by community paediatricians with special training in neonatology. However, they have busy clinical practices And so they pop into the hospital, and they see, you know, how's the baby doing and then they pop out. And they forget to discontinue the antibiotics when the swabs come back. So those babies in the regional hospitals are on antibiotics for ages. And if we could just get the nurses to prompt those paediatricians to say like, did the baby still be on the antibiotics because the swabs are negative	FGD-1	PN008
Resource Use	Hospital environment and resource use	Overuse of antibiotics	Umm But if I could have had the foresight in outcomes I would have... looked to try and measure things that were issues in the health system. And I think that like the overuse of antibiotic. An infection, I think, would have been one of them. We have the data it's just not great data and I would have worked a little harder on on getting that published.	FGD-1	PN008
**Core Area**	**Outcome Domain**	**Outcome**	**Illustrative Quotes**	**File**	**Participant Code**
Life Impact	Role functioning	Holding the baby	hmmm I was born in 1980 and 1980 there was not really family... family care so how they were involved as parents is what's not that much And...umm For example, they were not able to hold me. They held me for the first time when I was three and a half months old so....	FGD-2	PFN001
		Coping with emotions	it's also for the parent's perspective as how they view it They explained to me that ummm It could have helped them as parents as well to understand their own emotions or to cope with their own emotions as parents	FGD-2	PFN001
Physiological/Clinical	Physiological health	Infant pain	They were not able to give me some kind of comfort during um during procedures that were causing me pain, for example. Umm Yeah, so it was really an extremely different time as it is nowadays in most centres so Not everywhere. It's the same in the world, of course. Yeah	FGD-2	PFN001

Life Impact	Delivery of care	NICU design	That time, I understand our NICUs is very small. Each NICU has only a bit the number of NICU beds. It's around nine. So very small so Unfortunately, we do not have enough study space for families. so always we see very big One floor, one room for several infants. so, in that condition we are struggling how to actually engage the parent to treat or care then newborn infant.	FGD-2	PD004
	Delivery of care	NICU design	The design of the NICUs is really important because you want the family to feel comfortable and as PFN001 said siblings some of the older style units here that were built 30 old years ago in Australia are just one big massive room and there's no privacy. Obviously, there's not a lot of room around the cot for families.	FGD-2	PM001
	Emotional functioning/wellbeing	Family unity Siblings' confusion	So, a lot of the units don't sometimes have siblings even in there. So, some brothers and sisters don't meet their baby till they're home. So that's a whole process to start with. And then I guess for toddlers trying to understand why is mum and dad away so much	FGD-2	PM001
	Delivery of care	NICU design	So yeah, I think the design of the unit and having facilities that can support the parents being there or is really important. So, space, if we can, individual rooms, but also like facilities like you know somewhere to go and sit and relax and have some lunch And just to feel comfortable there.	FGD-2	PM001
	Emotional functioning/wellbeing	Parental satisfaction Parental anxiety	So, I think first actually First aim of family-centred care is actually first satisfaction of families satisfaction because they are always suffering they have high anxiety status so we have to provide the accelerate good condition for families. First step is I think at least satisfaction on families.	FGD-2	PD004
		Bonding	And second step, I think we need to increase the bond between infant and families. So, if they actually engage neonatal care.	FGD-2	PD004
Physiological/Clinical	Physiological health	Reducing morbidity	Final, actually… object should be actually improved outcome of infant maybe we can maybe but prepare some morbidities by introducing umm family-centred care. So final step should be actually we should improve we should prove improved the betterment of all preterm infant.	FGD-2	PD004

Life Impact	Delivery of care	Staff attitudes towards parental participation	Of course, both, but primary primary Staff attitude is very important. Because... I think. if we introduce maybe technology or new policy not always, old medical staff agree that changes. So, we have to convince them. But I think first step is attitude attitude of change of first provider of staff. And please, we change our policy, maybe. it's easy to accept for families.	FGD-2	PD004
	Emotional functioning/wellbeing	Parental trauma<br>Parental fear<br>Parental guilt	I think I completely agree with that again, PD004 because as a parent, you're completely quite lost. You're in shock. You're absolutely you know petrified and scared of this whole new environment and what's happening to your baby. Then you've got all the guilt feelings and all of those things and trying to still function as a family if you have other children.	FGD-2	PM001
	Role functioning	Parental knowledge<br>Parental involvement in caregiving (cuddling)<br>KMC	So, I think, yeah, we're led by the clinicians, by the doctors and nurses so unless you've been in a NICU you don't know what probably what kangaroo care is. It's not something that would be in your normal vocabulary. So, you know, I can't imagine, you know, a mum walking in on the first day after giving birth and saying, hey, can I kangaroo care? Unless she's informed, unless she's told about the importance of it. So I think, yeah, the clinicians, if they're leading that and saying, let's get bub out for a cuddle.	FGD-2	PM001
		Parental involvement in caring (holding)	Sadly, we still sometimes hear, you know, parents have told us that, you know, the nurse said they were too busy to get the baby out or there was too many tubes or too many things like that.	FGD-2	PM001
	Delivery of care	Staff education and knowledge of FCC	So, I definitely think, yeah, staff education on how important it is and making it a real focus will help the parents understand and then they can start to advocate. But if they... what we only know what we know, and we only learn that from being informed or being told.	FGD-2	PM001
		Staff attitudes towards parental presence	Yes, I think the attitude of the staff umm I think it's very helpful that day umm. they make it possible to feel a parent welcome in NICU	FGD-2	PFN001
	Emotional functioning/wellbeing	Parental confidence to	My daughter just turned 23 last weekend so She was in NICU and on CPAP for a few hours and then she was breathing quite well but Yeah, it	FGD-2	PM001

		communicate with staff	was, I remember I was just really scared having to ask what to do. And we are going back 20 plus years ago		
	Role functioning	Holding the baby	Because you don't get a second chance at that. Ummm Scarlett's then experience, she was in for four months She had lots of ups and downs. I think it was day seven when I finally got to have a hold of her and that just happened to be sitting in there on a Saturday night and a nurse saying, have you been holding her? And I said, no. And she was like, oh my gosh, she's seven days old. Why have you not had a hold? So that was the first hold I had of her. And we were involved in regard to doing cares and things like that.	FGD-2	PM001
Physiological/clinical	Physiological health	NEC<br>ROP<br>Holding<br>Parental fear	But then she developed NEC as well. So, then we got really scared to get her out of the crib and we were worried about that. So that was a big step back. As well, and she had quite a few different issues, retinopathy, surgery for her eyes, all of that.	FGD-2	PM001
Life Impact	Role functioning	Parental confident<br>Parental role	But I remember the big change with both the girls was when they got to special care, I guess. And I actually felt like they were in an open cot, And I could actually come in and kind of, I started to feel confident to pick them up and feel like mum. And I remember that was probably the first moments that I felt like mum it was hard to feel like real mum when they were in the crib and they were in the humidity crib and so reliant on you know health care to keep them alive so yeah, I wouldn't say it was a bad experience. We were involved but it was very limited in those first few weeks, especially with Scarlett before we yeah before I got to kind of fill that up	FGD-2	PM001
	Delivery of care	Hospital policy restricted parental presence and holding	of our parents how they uh how they uh yeah it was a completely different time, of course. There was no Ronald McDonald, so they could not stay nearby the hospital. And my parents already had My brother, who was only 10 months old still a baby when I was born. And…ummm they need to drive a long distance, one and a half hour driving so It was not always possible to see me that often and ummmm my mom she was able to see me when I was nine days old. So, it was for the first time and at that time in a NICU, there was not that much, so no kangaroo care.	FGD-2	PFN001

			so It was not always possible to see me that often and ummmm my mom she was able to see me when I was nine days old. So, it was for the first time and at that time in a NICU, there was not that much, so no kangaroo care. And I was also on the mechanical ventilator so that's That was probably the reason why could not hold me earlier. And I needed a long-time oxygen support so but as parents, they were able, of course, to see me, to visit me. It's umm what they could do was, for example just touching my head a little bit or my fingers or my but not really engaged in taking care of me so.	FGD-2	PFN001
		Parental involvement Parental advocacy	when I was transported to transport I think after three months to another centre that was closer to my parents' home umm And they saw the difference between those centres for the Academic Centre, they were able to do small things like touch me a little bit not holding but touching me. when I was transferred to the other, it was regional hospital. They weren't allowed to do anything at all. But my mom, she did not agree with that, and she was making sure that she wanted to do the same things as she was able to do within the academic centre. So, she really fight for that to get the permission and the permission or not she don't really ask permission she just She just really applied for it. And they were told that we don't do this with any parents. And she told them, this is my daughter, so I want to do this. And if you don't want other parents to see this, you just close the curtain. So, they won't know. But I want to be in contact with my own daughter. So, she really fights for it. But it was completely different time back then it's almost impossible to... to compare like it is now it is	FGD-2	PFN001
	Emotional functioning/wellbeing	Long-term detachment	But I think for the long term, I think I always, when I speak to other adults who were born in the same kind of period. And we... have a feeling that you were... don't really belong to your own family. So, it feels you have long for a long time you just felt like you were not really adopted but not really adopted you just were put in the family. And you don't really belong to each other. So, it's uhumm How do you say you feel sometimes lonely in your own family and it's uh That's what the other adults around my own age have the same kind of feeling. And I think it's because of not at that time it was not able to engage parents	FGD-2	PFN001

			that much. And there's a baby don't connect that well to your own parents. And I think the first months are really important to feel connected to your own parents as well. And… hmmm Yeah.		
	Delivery of care	Accommodation for parents	I think it's important if parents need to drive for a long time to make sure that the parents can stay as close as possible to the baby. My father, for example. I was transported for the regional hospital to the academic centre. He accompanied me in the ambulance because they were afraid, I would pass away. I didn't of course but I didn't It was in the middle of the night and he it was not possible to stay there in the hospital. So, he needed to go to sleep on a kind of a kind of it's not really a badge, but it was like a plastic chair. And then he took the first train home and it was, I don't know, four o'clock in the morning or five o'clock in the morning i don't know.	FGD-2	PFN001
			and I think it's important that when your child is born that parents need to be able to stay close to the baby and Luckily, we nowadays have Robert McDonald's The thing for the privacy umm to have, if possible their own room for a family with the baby. So, the mom can stay there with the baby. Umm a father can stay there as well with the extra bed there and maybe for if they have a child maybe just they could stay there as a family if that's possible but at least the parents should stay there with the baby very close but it's i know it's very expensive to build those facilities. But that would be the most ideal. And I think to engage apparent as much as possible.	FGD-2	PFN001
Life Impact	Role functioning	Parental presence	No, it'd be a perfect world, wouldn't it though where there was no separation and yeah, they could all be together whilst they're getting their intensive care. I think that's the Sweden model, isn't it like they have rooms yeah, it's Yeah, these names in Australia, everyone talks about Sweden	FGD-2	PM001
	Delivery of care	Sattf attitudes towards parental presence	our hospital still we allow parent to come to NICU until ummm 11 p.m. every day so after that because of maybe understaffing of nurses nurses usually don't want to actually around the parents come to NICUs.	FGD-2	PD004

		Scheduled visiting hours	So, of course. We are very happy to provide the Kangaroo care for or families Again, we have limited time and limited space we cannot afford every day or anytime so we actually have umm chat recording when and what day family can enter and do them kangaroo family care. So, we do not have actually only. we have only limited family can do kangaroo care maybe everyday rest of them rest of them 10 family can do kangaroo care. So, we limited we kept them maximum number for kangaroo care.	FGD-2	PD004
	Emotional functioning/wellbeing	Bonding<br>Stress reduction	I believe they are always feeling better learning doing kangaroo care and also Of course, we do not know the direct effect or in the infant but we believe it's better for them bonding as i said bonding between mother and infant so I experience it always satisfactory doing kangaroo care. Mother is very calm and also in fact, infants very calm. So, I believe it's a very good opportunity to, of course limited capacity but we want to provide kangaroo care as possible	FGD-2	PD004
	Role functioning	Parental confidence in caregiving	our nurses explain how to do kangaroo care and they have kind of booklet to show how to how to actually hold the infant and maybe couple of minutes explanation or more so family understand especially mother understand easily and hold infant on her chest. And always... they continue as long as or similar so. Actually, for families it's not difficult. It's a very actually natural behaviour or especially for mothers so maybe obstacles is our side. because we limited the number and also ummm time so families we believe kangaroo care is just natural. So, they do not see any problem to start kangaroo care.	FGD-2	PD004
		Increased Parental involvement	Kangaroo care was always a highlight for my husband and I whenever we got to go into the hospital, and you know they have recliner chairs right beside the cots. So, we would do that for hours on end as much as we could and as much as she was well enough to get out of the crib once again much more accessible and more comfortable in special care.	FGD-2	PM001
	Delivery of care	Staff engagement<br>Parental confidence in caregiving	It is a little bit awkward with all the tubes, but the nurses were always really great in getting us comfortable and getting in a spot where we could just, yeah, sit there with her on our chest for hours at a time until you got kind of dead arms, I guess you know you would stay there as	FGD-2	PM001

			long as you possibly could. So that was always positive always enjoyed once I felt confident in doing her cares.		
	Role functioning	Parents' involvement caregiving and stay close in their infants helped expressing breastmilk	So, you know, cleaning her eyes and her mouth and, you know, changing nappies and things like that, feeling involved and engage was really important expressing breast milk ummm That was a challenge for me, but it was always better if I could do it by the cot side. I was a bit more successful that way. So being close with her at those times.	FGD-2	PM001
Life Impact	Emotional functioning/wellbeing	Bonding	Beautiful. Absolutely. Like, you know every mom loves to hold their baby and to do that while they're so sick and vulnerable is really special and yeah, and starting to get that bonding and yeah it was just a sweet precious moment that I don't think you ever forget those moments.	FGD-2	PM001
	Delivery of care	Parental knowledge in infant care<br>Parental confidence and preparedness for discharge	And one thing I always said to people and still do is the amount of education that I received in the NICU. So learning so much about how to care for her I can't imagine going home after three days say in hospital after having a baby it must be just so overwhelming and feeling lost because we took our girls home on four hour feeds you know they were quite in a routine but I felt so educated on how to look out for her like we did a resuscitation course before we went home I really did value the education that I received as a parent in the NICU as well that was really valued.	FGD-2	PM001
	Emotional functioning/wellbeing	Coping with situations<br>Bonding<br>Anxiety	And I think dealing with emotions as parents to bonding with your baby and holding your baby that's One of the things you can do as parents and... it gives your baby comfort. It gives yourself comfort and I think it lowers your own anxiety.	FGD-2	PFN001
	Role functioning	Parental role	as a parent, you feel in the beginning, I think you feel you are not able to do can do a lot when you are in an NICU for the very first time but actually you can do so much for your baby And even the tiny things like taking care of your baby, it will help you. you feel like a parent. You don't feel like a visitor	FGD-2	PFN001

	Delivery of care	Shared decision making	Engaging parents in decision making as much as possible. Of course, not everything is possible to sometimes they should maybe rely on the advice of the doctor. But I think in a lot of cases they are really involved in decision making process. And I think in some part, maybe they should You know, it's so many things they do so many things Some things are ethically it's a bit difficult because with parents you can You have to look for the benefit of the child and sometimes as parents you fight a little bit too much. Because to keep the baby, but sometimes it's maybe sometimes better to to let the baby pass away because of the long-term consequences or the short-term ones.	FGD-2	PFN001
			So probably we have... a different character. Most of the time of course we provide true information for decision-making of medical treatment. But Usually,... hmm more some family photo our suggestion for even our decision of course they have sometimes ummm they have a definite opinion. But finally, after that most of family try to actually forwards. hmmmm It is... professionals' suggestion or decision. So, of course. they always say they are they don't have enough medical information so doctors when nurses say this to usually trust your opinion so most of the time maybe specific for Japanese.	FGD-2	PD004
		Parental knowledge	It's kangaroo mother care, it's education for the parents on what's important around you know breast milk, around you know bonding time, kangaroo care ummm infection control, hygiene, it's around so much that they can learn to give their baby the best possible chance	FGD-2	PM001
			I mean, we hear a lot around, you know, third world countries or poorer countries, you know, Vietnam and things where they're doing a lot of great family centred care initiatives and they're putting those important things around the family connection and care and doing what they can within the environment they're in. So, they might have a room where there's 50 women all in one room and babies.	FGD-2	PM001
		NICU facilities	For me, it's about education, connection or you know bonding with the baby and but to do all of those things you need to have the facilities. So that's letting the parent feel comfortable.	FGD-2	PM001

			So it's accommodation in Australia is a big issue because we are a very big, vast country and you know babies that are born you know it could be six or 12 hours away if you're in Central Australia from a hospital. So families are having to transfer you know their whole lives at a huge financial cost to themselves a lot of the time. So families are having to transfer you know their whole lives at a huge financial cost to themselves a lot of the time. So yeah, probably said more in that than what you wanted to capture, but I think it is um um a range of things that can really bring the family together with hopefully reducing the stress and financial burden on the parents so that they can be there and can be parenting and feel confident and umm give them the best chance to succeed and you know go home as a happy family unit	FGD-2	PM001
Life Impact		Parental knowledge in infant's care and treatment	Because we know some of these babies will have ongoing issues in the short term and the long term. So, if we can help those parents be prepared for that and ready and aware of what support and interventions are around for them, then I think that's trying to make the best of a really tough situation that they might be in.	FGD-2	PM001
			I think it's just uh providing that the family all the tools and information they need ummm during the NICU stay and also after the baby go when the baby goes home, and the parents know how to take care of the baby.	FGD-2	PFN001
	Role functioning	Infant-family relationship	a short time out to come. it's uh probably relationship between infant and infant their families. So, I think it's a... kind of short-term outcome. So, if we do family centred care maybe relationship ummm getting much better them	FGD-2	PD004
Physiological/Clinical	Physiological health	Infant neurodevelopment	... I think final final study outcome should be the improved development. Of, especially a preterm infant. Because... we are doing actually we are doing very bad things to actually prevent natural neurodevelopment of preterm infants. but family centred care may cover some of that minus effect so short-term is relationship, final outcome is improved of infant neurodevelopment. That's my opinion.	FGD-2	PD004
Life Impact	Emotional functioning/wellbeing	Mental health of parents	I think it is I think overarching would be that the family unity is going home .... in a good mental health space, I think the children have a better chance to thrive if the parents aaa You know if you once again, feeling educated, confident, informed.	FGD-2	PM001

	Role functioning	Parent organisation support Empowerment parent	I think all NICUs should have a good relationship with their parent organisation in their country or their region. Because as we know, sometimes you go home and you kind of feel quite alone and you don't have all that support from the hospitals anymore. So, making sure that parents know where they can go to after discharge for further support and, hmmm you know, parent organisations are great in that regard, in guiding them and supporting And sometimes providing services as well	FGD-2	PM001
	Emotional functioning/wellbeing	Breastfeeding	Yeah, and hopefully they go home with you know increased breastfeeding and all of those types of things.	FGD-2	PM001
		Bonding of infant	Because... if you have... well family-centred care, maybe you will see differences in mental health in the preterm or the babies who were in the NICU. So, umm and maybe you will see differences in bonding with the parents.	FGD-2	PFN001
		Anxiety of preterm in adult life	I think... I can understand that when you have over has family-centred care that you will see maybe differences in outcome in the being emotional stable when you are growing up or hmm maybe have less anxiety or less anxiety or hmm as the one who was born preterm.	FGD-2	PFN001
Resource Use	Hospital environment and resource use	NICU cost savings	hard to prove but I believe if we introduce family-centred care maybe cost for running NICU getting less. So, I think if we okay, I want to include economical.	FGD-2	PD004
		Readmission	I think on top of that as well to just add to that is reduced hospital rehospitalizations you know if those parents are educated and informed and you know how to you know um sterilize bottles properly and do all those types of things then you know hopefully you're going to have lower re hospitalization	FGD-2	PM001
Life Impact	Emotional functioning/wellbeing	Birth trauma Separation trauma	And I mean, obviously birth trauma and delivery was very different. But what we saw with neonatal families, a lot around separation and that kind of trauma and not being with your baby. Yeah	FGD-2	PM001
		Parental stress	the stress of not being able to be with their baby as much as they'd like to and all of those other things we talked about, you know, travel, accommodation, finances	FGD-2	PM001

Core Area	Outcome Domain	Outcome	Illustrative Quotes	File	Participant Code
Life Impact	Role functioning	Parental involvement	when I ummm admit my child and I was in the NICU in 2001. I didn't feel like I I I I didn't feel like I was a mother because the the in 2001 they don't allow me to do anything in the NICU in Portugal. In that NICU Because my daughter had a lot of problems and she was in a risky a very high risky situation. She had several pneumothoraxes, three, pneumothorax in this first 10 days. She was with 500 grams in because she'll lose weight, and they and I didn't feel like a mother. And I was suffering, And I was seeing my my child suffering as well.	FGD-3	PM004
		Parental knowledge infant’s care and treatment through parent organisation	I asked my brother to search for all the information to help me to understand what is the the immm viability of the child what we can we has parents that doesn't know anything about prematurity issues what we can do. And I read a lot of things But of course, it was papers and information hmm scientific information that it was very difficult for parents to understand But umm I had the opportunity to read a lot of things from Bliss. It is... umm association organization in in UK that has some some information for parents	FGD-3	PM004
		Parent advocacy to involve their infants' care	I had the opportunity to read a lot of things from Bliss. It is... umm association organization in in UK that has some some information for parents And because of that, I started to negotiate with doctors and nurses to let me do something to to create hmmm bonding with my child,	FGD-3	PM004
	Emotional functioning/wellbeing	Bonding	I started to negotiate with doctors and nurses to let me do something to to create hmmm bonding with my child, I understood at that moment that my voice is important so I I talk with my daughter and hmm step by step they introduced me in the care of my child.	FGD-3	PM004
	Delivery of care	Shared decision making	the parent in the lead is very important to do also with the daily rounds with the doctors to let parents tell them how they, umm if they have new observations about their and baby	FGD-3	PN006

	Role functioning	Parental presence	the parents should have access to the baby 24 hours each day and they are the primary caregiver.	FGD-3	PN006
	Delivery of care	Staff communication with parents	I also think that as a nurse I need to umm have an approach where I ask the parents, what can I do for you today. So, I shouldn't go into the room and say, no, it's time for this and that. I should ask questions.	FGD-3	PN006
	Role functioning	Parental presence for 24 h access<br>Parental role<br>Family unity	I think the most important is that the parents must have access to the baby and they should be the primary caregiver and I should support them both parents and sibling as well family-centred care for me is also person-centred care where the family If the child that I care for, the parents and the siblings.	FGD-3	PN006
	Delivery of care	Shared decision making	family-centred care means involving the parents in decision making or also in the care of the the infants and also to a large extent from African perspective also involving the extend family because in Africa whenever a child is born everybody is happy they wanted to see the baby you know. So at times... umm There are some... umm umm Why should I put it? For example, if your child is being admitted to the NICU for a longer period, there will be some rumour in the community like aaa She has given me a sick child	FGD-3	PN010
		Hospital policy	because of lack of space in our NICU. you don't allow... we don't allow apart from their mother and the father, no one is allowed to the NICU. Yep.	FGD-3	PN010
		Parental knowledge infants' care and treatment	Yes, especially for adolescent mothers and new mothers And we got... hmm that's the first time they are giving birth and they don't know anything about motherhood or parenting.	FGD-3	PN010
		Staff communication with parents<br>Staff reassurance reduces parental stress	So I think... hmm explain ... the condition to them and also umm reassuring them. Giving them reassurance as well as also be empathetic ummm showing empathy towards them because um some of them become confused and also very worried anxious.	FGD-3	PN010

	Role functioning	Friend support	I also think that we need to let the parents decide who's important for them to be with them at the NICU because we always say that the parents should be at the NICU but perhaps for for the mother her mother or a friend is more important for her than the father at the time. So, I think we need to ask them and let other person than the parent visit the NICU.	FGD-3	PN006
	Delivery of care	Parent-staff relationship	Professionals training to understand what kind of parents are are there. because we are different from each other and some some of them are because ... the several things that I observe when I was with my child in the NICU, was some stressful stressful situations because of the the different kind of parents and different kind of nurses and doctors umm. Because we are different and some of them are some of us like to to are so so people that are so organized they they have plans and they do it everything in the in the same at same the same the bus at the same hour the you know the feeding is this And this creates sometimes stress situations in the NICU.	FGD-3	PM004
Life Impact		Continuity of care	it is the we have um the we is is women being are different. Sometimes, you know, the the parents when they they come to to the hospital, they they are expecting that some rules or some behaviours are all all day the same for instance for example And others don't care about it. They just want to that the child is being caring by professionals for the best way. But this creates stress.	FGD-3	PM004
	Role functioning	Bonding through KMC	yes, to get um umm bonding as soon as possible with kangaroo care or ummm to get parents involved in the whole process during the day.	FGD-3	PN005
		Parental involvement in daily care	So, with the daily rounds with the diaper change, we're doing the care, gather information that is all components that are very important for family-centred care. but also, in normal life	FGD-3	PN005
		Parental role	So, it's not specific for the NICU, but umm umm explain why you do what you do. And... ummm Yeah, get parents in the lead and their families.	FGD-3	PN005

		Holding	a nurse holds the tubes Because it was like that that they do it and then I I had my baby for some seconds to put again her and get the duvet. This is the first thing that I have done And what was important for me is that I was only the only mother that do it	FGD-3	PM004
		Peer Support	It was a space with 12 NICUs And no other parents do anything. So I asked them, I asked the NICU if I could share that because I was do it alone with my husband also but my husband was working so he was not there all the time and when I I understand that the other parents look at me. and They don't do anything else and I asked the director if I could share the information that I get from my brother to share with the other parents for them to study that. And if I could help, of course, not me, but with the nurses and the doctors, help them to do it if they want to to understand that this is important for parents.	FGD-3	PM004
	Delivery of care	Parental satisfaction with communication	And that's why they invited me one month and a half, my daughter was three months there. But one one one month and a half when one month and a half after this beginning to do this because they allowed me, they taught me. And I am so grateful for because they listen to me they understood that this was very important for me. They allowed me to share all the information and in more weeks all the doctors, parents working with doctors and nurses in this kind of little steps of care they done. That's why they invited me to create because we change the environment of the NICU. It was very important. and um And I think this is one of the things that is that I'm always telling that we should listen parents also we should of course the now we know more the investigation the research and the results of the application of an infant and family-centred and developmental care is supported in the evidence-based	FGD-3	PM004
		NICU design restricts Parental presence and involvement in care	we have Kangarooo mother care. And as I said, we have a small, no, it is a teaching hospital. We have very small NICU.I think our capacity is not up to even 10. It's a very small NICU so usually it's the nurse who do most of the work because we don't have space for the mothers to come in. So mostly is the nurses who	FGD-3	PN010

			provide the care. The models only come in For example, I mean, it's time for when it's time for them to breastfeed come and breastfeed their infant and also those who are doing area for Kangaroo mother care we don't even have a space for them. So those will come to the neonatal we need to do the kangaroo mother care		
Life Impact		Staff coaching parents	In Africa you have other conditions, and you have other problems than we have for example in Sweden, we always have lack of space sometimes. But I do think when I work the most important for me is that I would like to cooperate with one of the parents and not with another nurse or assistant nurse. I would like to work together with one of the parents and if possible, I would guide the parents to care for the baby themselves together. And that, I think, is important because as you just told us, PM004 otherwise if you can't understand that you're a parent and that the child you care for is yours, so I think that's so important.	FGD-3	PN006
		Staff attitudes towards FCC	it's very, very good that I understand that now it's not like that so involve parents of course we have some issues because we have different inequalities between the the spaces and the different hospitals in Portugal. Some of them are more new hospitals that have a ... better places and better equipment but we have all the hospitals that don't have so much so much good infrastructure in the NICUs. but We have a very committed professionals in Portugal. This is very important, very committed very very good professionals and They try to do the best in the infant and family centred and developmental care but of course that we have umm we have... ummm more, how can I say, we can do it better of course we could do it better.	FGD-3	PM004
Life Impact	Delivery of care	Staff resistance towards FCC	we have to manage is that ummm we have some nurses who experienced that they aa experienced that when they don't feel the baby they don't have enough information aaa to... do the daily report of they have to feel the baby they have to wait it by themselves because otherwise they have a lack of information	FGD-3	PN005

			and that is in my opinion, not true because you have always had the parents who can also give a report about the about the baby that is a kind of a learning curve hmmm for our department over the last year. Because some nerves, well, the parents ...they steal my work. But well, your work is changing.		
		Staff education about FCC	So when you are aware of this and aaa aaaa have with your team discussions about what is necessary and what is the latest research about uh aaa the parent in the NICU. Aaaa Well, you have to be aware of it. .... I guess that's the biggest challenge to get everyone involved.	FGD-3	PN005
		Visiting hours to involve parents' infant care Scheduled Parental involvement	Yeah, we have... inadequate space, yes. So, ummm ummm , we don't have a place for the mothers. So...ummm for example um mothers who have gone through ICUs gained a maternity ward too many staff for them how to work for the maternity comorbidity to the NICU to breastfeed and go back intermittently. And also. Ummm I said, ummm some infant admission at the NICU have been cut short because of equipment issues So... they are being transferred to the neonatal unit ward . For example, those who are ummm I'm going to physiotherapy. They have been transferred to the university ward to continue their um sorry phototherapy continued their phototherapy. We had the phototherapy in the aaa in the paediatric ward. Yes. Aaaa And we had a lot of mothers who we have a lot of admissions. So our time to we do transfer some to other teaching hospital Yes.<br>Jos Latour= So would you have your parents I mean you mentioned that the mothers come in for breastfeeding<br>PN010= Yes.<br>Jos Latour= If I challenge a little bit, are you having 24 seven hours I was having parents allowed to come in or are you saying Or why don't you come during breastfeeding? I'm just curious. What?<br>PN010= umm We have scheduled for them. we have scheduled for them and they should come in and they should go out	FGD-3	PN010

Life impact		Lack of accommodation option hinders Parental presence	24 for seven years the full seven days a week the law says that they can. But they don't have conditions for them to be there you know. Some of them have some rooms, but not near the baby. it's outside the NICU, outside the NICU. Umm But they don't have conditions for like a bed for parents bathroom for for them to do their hygiene. But the law says that theyummm should allow parents to be there 24h for seven days only in only in like a several situations that they could they couldn't they could not allow them. But the conditions are ummm not the conditions are not they don't have it so so they don't stay there. Yeah.	FGD-3	PM004
Life impact	Delivery of care	Staff attitudes towards parental presence	In our ward, we allow parents for 24 hours, but we have one bed aaa for one person. So, some parents choose to sleep together at home or in a Ronald McDonald's house but other parents sleep with our infant and there is also some nurses who don't support it because they say For instance, well, it is not this is an ICU with a lot of alarms And it's not very dark in here. You should rest and make milk Shall sleep with your husband at home? Hmmm And that is also the same learning curve as I mentioned before. Because when your child is older, you would never leave your child, I guess. So why cant you sleep with your infant when it's in the incubator So, um umm but... parents our welcome 24 hour a day and that we don't have a law that say something different.	FGD-3	PM004
Life impact	Role functioning	Parental involvement in infant care	I guess hmmm the kangaroo sessions would be uh uhhh because it is very you can make it objective ummm And maybe the... That is for the parents. And do you want outcomes for the for the baby or for...parents	FGD-3	PN005
Physiological/Clinical	Physiological health	Infant sleep pattern<br>Infant stress<br>Infant weight gain	Yeah, for nurse, yeah, there are so many so many things you can think about.ummm ummm I would say what was, but this is more qualitative instead of quantitative Ask the parents how much impact they had on the care for their children And if they felt they were hurt they were ummm that they could have... ummm that they are a part of the daily care so for instance But	FGD-3	PN005

			you can also you can also have things for the baby like the sleeping pattern the comfort of the baby the stress or... ummmm weight gain ummm that sort of thing but I I can I can point great one important thing.		
Resource use	Hospital environment resource use	LOS-NICU	the government the the what could we also the outcome for for instance could be more less days in the NICU or the charge was is earlier if we could implement a true infant and family centred and developmental care.	FGD-3	PM004
Life impact	Role functioning	Less Parental stress<br>Less Parental anxiety<br>More Parental confidence in caregiving skills<br>Improved family functioning	so for the parents is the I think it's the the one outcome that is immediately, I think, is they're comfortable they're the stress the less of stress less of anxiety more confidence when the child is discharged because they are more confident that they they will do correctly the take care of the child when they go home so. and this could reduce also the stress with others the other siblings, the the way they they behave with the other the other the brothers and sisters of the baby also when they are have more more trials.	FGD-3	PM004
Physiological/Clinical	Physiological health	Weight gain<br>Breastmilk<br>Breastfeeding	And also for the baby is the baby the bonding also that the baby creates and that gain more more weight. PN006 was telling the breastfeeding because the baby is near the mother. So the the milk is is the mother could have more milk and this is very important for the baby, the mother milk. Also could be the breastfeeding the because they do more kangaroo so is a good outcome that all the the ummm ummm ....how can i say the ....the the pressure, the heart pressure the the the breathe everything is better if is if the baby stay more time doing kangaroo care you know we know that it's is a very good practice.	FGD-3	PM004

Life Impact	Delivery of care	Parental satisfaction	and then also the nurse and the doctors because they the involvement to involving the parents they also think they also they will understand the benefits. Also, in the with the parents and also with the baby so the doctors and nurses become also I think more satisfied to do it and to to do it to understand it that brings benefits for all of the team. because it's a team all of them are a team and the costs.	FGD-3	PM004
	Physiological health	Better adult social life	And the long term is the sequels because we know that reduced is different kinds of sequels also I read the name … Dr. Dieter share in the last EFCNI meeting also the benefits with this because they studied ummm a lot of babies in different groups one after that they have more ummm ummm participation of the parents and the others that don't have. And they understand that the social ummm ummm the way that these kids and and um ummm and adolescence that was born premature ummm behaved with the other groups are the social socially socialization are better are better in that study. When they have more, they had more infant and family-centred care, more contacts we did the parents they are most socially socially. I cant sorry my English, but they socialize more more better rates in during the high school or the the school. Because they have more impact when because they had more time umm with with parents and did kangaroo and socialization. If they have more contact with the parents in the NICU, is some of long-term outcomes that they understood in the first ummm hmmm the first the research in this area about also aaaa cultural outcomes yeah.	FGD-3	PM004
	Emotional functioning/wellbeing	Parents mental health<br>LOS-NICU<br>Shared-decision making	For me, I think measuring the psychological way of parent is very important because I have to reminded that um parents ummm just transitioning into um and post NICU. you are being asked to…aaa send your child to the NICU. I think they have a physiological impact on them severely and also the duration will stay of infants in the NICU is also very important as our environmental parent in decision making.	FGD-3	PN010

	Emotional functioning/wellbeing	Staff workload<br>Staff burnout	And also ummm also measuring the workload or burnout among staff because working in the NICUs is really um demanding yeah.	FGD-3	PN010
Resource use	Hospital environment resource use	LOS	there are important thoughts in parents experiences that we need to capture in some way length of stay, for example, is one way could we reduce the time at the hospital if the parents are taking care of their baby from the very beginning for example.	FGD-3	PN006
Physiological/Clinical	Physiological health	Oxygen support	And also, I think, um umm ummm medical evaluation of the the baby umm time with oxygen and so on. But I think that it's not easy to find it's it’s a different it's a difficult question Cansel to answer.	FGD-3	PN006
Life impact	Delivery of care	Staff attitudes towards FCC	staff attitude towards parents is very important	FGD-3	PN010
Life impact	Emotional functioning/wellbeing	Parental satisfaction with NICU experience	I didn't want to use the word satisfaction. Yes, because... no one for me no one would be very satisfied at the first instance to send your child to the NICU. No one will be satisfied at the first instance. Yes And even throughout your stay in the NICUs you know For example, should I say there are different kind of nurses that parents work with And they are not going to treat you the same. At times you have the good one you meet the good one you meet the bad ones I think. So no parent would say like a half egg a quality stay in the NIC. From my experience, yes, it's a mixture of both quality.	FGD-3	PN010
Life impact	Delivery of care	Staff communication with parents	The thing is sometimes is the way because they are pragmatical they are more rational or parents are you know that's why I told that it's very important to know the parents to know parents and to know us. Because I'm working, I'm a director in my bank and I have several teams. And I needed to adequate my way to speak with my team with the different personalities that I have in my team for not being a bad person because the people needs information in different in different grades and scales and with	FGD-3	PM004

			different words someone are more pragmatical and I could be directly to the point But others are not so like that. So, we need to equate our communication. But of course, the ones that I put in the red one, they are not bad. They are very good professionals. But sometimes the way they speak with me was bad for me. But not because they are bad person.		
Life impact	Delivery of care	Parents satisfaction with staff attitudes	But I think no one wants to start that parenthood at the NICU, no one wants to do that. But they could be as a parent, I think you could be more or less satisfied when you say goodbye to the NICU. And if you are more or less satisfied depends on of working there. And I think that's what you're telling us PM004 right now.	FGD-3	PN006
Life impact	Delivery of care	Parent-staff trustworthy relationship	I think also that's where... effective communication skills come into play is how you talk to the parents will let them know how ...how much you care or care. Yeah.	FGD-3	PN010
Life impact		Parental involvement through HCPs engagement	you can't just talk to them you need to involve them Yes. And they must feel that they are involved in the care of that child.	FGD-3	PN006
Life impact	Delivery of care	Staff reassurance	Even if you are not even allowing them into the NICU, even just one statement will just come down like we'll reassure them like my child is safe so yeah.	FGD-3	PN010
Life impact	Delivery of care	Staff cultural competence<br>Staff communication difficulties due to language barriers	Yeah, and what makes it also different umm ummu with the interaction with parents in the communications with parents is that you also have culture differences, language barriers and ummm that makes it sometimes difficult to ummm really ummm point out what parents need in what state they are and ummm what kind of information they need. So maybe sometimes I am placed in a red box. Because there are some culture or language barriers.	FGD-3	PN005
Life impact		Parent's culture affects parental involvement	Yeah, that's very difficult because ummm we don't have we don't have information. I guess we don't have enough information to get into different cultures. For instance, we have some ummm Chinese parents. And in that culture, it's very	FGD-3	PN005

			normal that the mother has to stay at home. And the father is coming to the NICU. And the mother is not involved in any care. Just bumping milk. And in my opinion, that is not a good idea. We have to get the mother here but then the father gets angry and says. No, in our culture, we do it like this. So, we cannot force to bring that woman into the NICU. We only can explain what we think it's important and they can do with the information what they want. So, the culture differences it's important to take into account, I guess. And it's just an And...ummm .... Yeah. It's just... one thing I want to mention here ... that you cannot force to bring the parents in Even if you think it's important.		

Core Area	Outcome Domain	Outcome	Illustrative Quotes	File	Participant Code
Life Impact	Delivery of care	Staff education and training about FCC	The best experience that we had, it is very, aaa I mean, it happened just this month when we started to to educate, to train the staff of the private aa maternal aa private NICU. Aaa They had no idea. I mean, no, directors that they invited us to to do this training. I had the experience. The NICU has two directors and uh One of them aaaa has worked in the uk for several years. So she has the experience of family-centred care And the other one has worked in Cyprus close to a developmental physiotherapist who practices aaaa the family-centred care so She also had this experience but the staff had no idea I mean all the staff. Neonatologists, nurses, midwives everyone. So we started from zero, from point zero And aaaa aaa for me, aaaa the best outcome for this was that aaaa They were shocked. I mean, they had no idea. they realized that aa the way that they were hospital they they practiced their job till now was totally wrong I mean uh They had no idea about very basic things in family-centred care, meaning developmental care because family-centred care has very a key component is also the developmental care. It is not just to cover the sentimental needs of the family. I mean, and the rights because it is human right to keep the baby close to the parents	FGD-4	PM002
Physiological/clinical	Physiological health	Parental sensitivity and responsiveness Sepsis	I feel that we succeed when we see the parents respond to the baby's cues every time and we see that there is something that we see And we also knew that we succeeded when the parents brought late onset sepsis before we even see it in any tests. And that means that they know they're a baby. and then they can yes read their cues read their signals and respond to them	FGD-4	PN007
Life impact	Delivery of care	Parental autonomy	And it is also important In my opinion that they do it on their own initiative and so it's not the nurses or the doctors that facilitating initiative regarding the baby aaa for some care task that the parents do them by themselves. And that also means that we have feeding hours every second hour or every third hour and we.. And it's okay to break the rule for half an hour if the parents see that my baby is actually telling me that she's hungry. And then we feed the baby. Or they feed the baby. No, we don't. They feed the baby on their own initiative. And I feel that that's when they're... the parents are	FGD-4	PN007

			engaged in the discussion and they do decision making themselves to feed the baby and do not involve us.		
Life impact	Delivery of care	Staff communication with parents	the challenges and difficulties with staff especially uhhh currently on the NICU with nurses. So on one side, they have to struggle with the aaa daily work and then on top working with parents and in Germany um but at least in Berlin. it's we become more families with refugee or migration background and then the communication gets really difficult because normally you need aaaaa a translator uh so we don't have them in the world 24 hours and so on. And I can imagine because uh how the nurses feel because when I talking to parents in a calm situation when they are still pregnant and trying to explain the difficult medical things than to imagine how to explain them when there's a little bit stress because there's alarm going peeping and so on and the parents are afraid of it.	FGD-4	PD002
Life impact	Emotional well-being	Parental anxiety<br>Parental advocacy<br>Parental confidence	On the other side, it's a positive experience and working with EFCNI and I and seeing Also, my parents when they leave the unit with their baby and seeing the face on them changing from the pregnancy where of course they were worried about how it's developing what is the outcome seeing them at the delivery where the anxiety was maximum. I guess, in the first one or two days and then to become advocates and specialists for the needs of their parents so it's as a more or less outsider you can see how will they change and this is for me a really interesting positive experience if those parents which they are just that we were afraid of all this medical system, then fight for their kids and say, no, no, no, I don't want that. And it's very interesting. And also if you are on the ward and at the very first moment they are afraid from all these alarms and machines and they get adjusted to it. It's still, of course kind of annoying and and baby... the parents will get afraid if there's an alarm. But they get used to it and there's casino that then they say, no, I see my baby is hungry and I will do it regardless of what you said or whatsoever. So, this you can see if you have a family-centred care if the	FGD-4	PD002

			parents are allowed are available then they become more self-confident uhhh and you will have, of course, a quicker and better result at the end.		
Life impact	Delivery of care	Staff communication with parents Staff attitudes towards parents Parental understanding	Yes. I completely agree umm what sometimes you also talk about aa confi aaaa communication PD002 and what I think is when we are challenged them family centred care, that doesn't always mean the organization, even though we have family rooms there is something about the culture and something about the individual approach that we all have when we all are taking care of the babies and their families and their families. But sometimes I can go to a room, and I think. Why are they just in their bed and they're a baby in the cub? And the parents are watching TV, why don't they have the baby skin to skin? It's a clinical stable 30 weeker. It can be skin to skin. You are there. We have talked so much about it. So is there something in our communication that we do not give the right information. Or do they understand our information in the wrong way or what are the priorities of the parents? and when you start questioning and asking and being curious about why the baby is in the cub. Well, they are too hot when they are skin to skin. Oh, bed coin would then put little uhhh what do you call it uh English something in between the skin of the baby and the skin of the parent just a little so you have some skin to skin and some to take out the wet, the silk of the sweat so it could be comfortable for everybody. Well, there was a solution so that was why they didn't have maybe so much skin to skin. So, it's something about communication and how you communicate. I'm being curious and instead of saying just every morning, oh, these are the parents	FGD-4	PN007

			doesn't want to make skin-to-skin. Okay, there was a reason about. So, look for also look for the family resources and although the wrong findings. You know what I mean? So be a resource finder as a clinician instead of a umm negative finder. Does that make sense? I don't have the English word right now.		
Life impact	Delivery of care	Parental knowledge infant care and treatment	Yeah. And something else, I think that we have, no, I believe that we have to explain to the parents the benefits of ummmm every action of their action in the NICU, let's say for the kangaroo care is not only enough to tell them uhhhh that I mean, the benefits of the kangaroo care that they could they can see also in the internet I mean thermoregulation and aaa palpitations and this and that that okay but also to give them in simple words the explanation of the mechanism why this is happening. In order to make them aaa to participate ummmm how to say to to raise their commitment is that because they What I have seen even in the the NICUs that they practice some kangaroo care they do kangaroo care and they take selfies or they they are talking to the they have the baby here but they are talking to the I mean uh through the mobile to the family at home etc because no one has explained them that it is the perfect tower the the golden hour for them and their baby.	FGD-4	PM002

Life impact	Delivery of care	Staff communication with parents	To do all this, to speak, to listen to their voice to they do not even know that they have to keep the eyes of the baby away from direct light during the kangaroo care. Or I mean, things like that because they have no idea how it works. Aaaa So maybe during the education Not maybe. I'm sure that there is a need for that, that we have to give also in a simple way not very scientific and very complicated but in a simple way the explanation the the mechanism of every action that they are doing aaaaa in the NICU in order to to participate in the right way I mean to do that correctly and to because even And the outcome is the best but also their satisfaction is the best because they they realize what they are doing.	FGD-4	PM002
Life impact	Delivery of care	Staff communication with infant	But you know what I'm talking about So even and all the other things, I mean, I have seen even the nurses in Greece. When they feed the baby, they are totally disconnected with the baby. They are feeding the baby through the tube and they are talking to each other. They are looking you know They do not watch what they are doing. They do not communicate with the baby when they feed the baby and because they are disconnected they that's why they need also treatment they need also to know what they are doing and aaa how they have to do that Or they feed the baby.	FGD-4	PM002
Life impact	Delivery of care	Staff education and training about FCC Infant distress	In the level two this way from the neck you know like this and like this and the parents that they are watching. They're doing the same so suddenly I enter the level two ward and all everybody nurses and parents and mothers, they are feeding the babies like this with a bottle you know you can see you can imagine the and that was an open ward for the parents. The parents were free to enter any time to feed the babies anytime during the day and the night. But what is the benefit of this? If they do that in such a traumatic way for the baby And they provoke gastric pain to the baby in this way of feeding. I mean education staff staffing um do we have enough nurses.	FGD-4	PM002

Life impact	Role functioning	Parental role<br>Parental confidence	I mean, it is aaa parents also is an obstacle that we have to to face we have to I mean, it is a challenge also that it was a surprise for me. I thought that all the parents will be ready to I mean enthusiastic and let's go and yes this is our role, and we would like to do that and that. But this is not the reality in Greece. Parents<br>are afraid to take responsibility they feel they do not have self-confidence. They feel that they do not know anything. And maybe they be harmful if they interfere with the babies and...ummm This is what we face now	FGD-4	PM002
Life impact	Role functioning	Parental role	Oh, okay. If I want to test or evaluate intervention. Aaaa I think... the most important part for me is that the family or the parents felt that they made a difference for the baby. that they were important that they felt important for the baby during the admission.	FGD-4	PN007
Life impact	Role functioning	Parental involvement in their infant's care	That's my own hypothesis that that there are also different levels of expectations of how to feel being involved because when the organization and the NICU is decorated like it is in Greece. maybe the parents have lower expectations to the level of involvement. when there are not person as much because they do not fit into the room because the clinicians also needs to be there in case of adverse events and treat the babies. And I think the expectations from the Nordic or the Scandinavian parents they are quite higher than in Southern Europe. And I don't know how to take that into consideration when developing the core outcome set that you want to develop. So I don't know what to do yet.	FGD-4	PN007
Physiological/Clinical	Physiological health	Breastfeeding	its discharge criteria feeding at discharge, breastfeeding priority above bottle feeding.	FGD-4	PN007
Resource Use	Hospital environment and resource use	LOS<br>Readmission	And the length of stay and also readmissions.	FGD-4	PN007
Life impact	Emotional functioning and well/being	Parental self-efficacy	And then you could use the parental aa You know, the caring, something like that the self-efficacy of the parents when being discharged.	FGD-4	PN007

Life impact	Role functioning	Parental role<br>Parental satisfaction with their role	the problem you raised, of course, is different expectations from the bottom there might be different expectation from the parents you could get along with that if you say how satisfied were you with your role? So, to say, okay, even if you were not 24/7 in the room how was it how was it your role, have you been satisfied well all your concerns and expectations to be fulfilled. I think this is one ummm possibility to make an outcome for the parents um just and I also would assume if you have parents was satisfied with the role, they would even accept a slightly was an outcome for the baby because they were able to do everything and they recognized, okay, it was maybe the max ummm we could get out of this situation.	FGD-4	PD002
Resource use	Hospital environment and resource use	Healthcare cost	the medical thing and for the politicians. aa Of course, we need long-term outcomes to show them again and again and again if you start early on your health costs will decrease dramatically. if you do that and if the babies do better, you avoid long-term complications. Things like that.	FGD-4	PD002
Physiological/Clinical	Physiological health	Breastfeeding	And regarding the outcome of nutrition, I completely agree, PD002. But regarding the outcome of nutrition, breastfeeding is very there's a bit vulnerable outcome said because We know that the milk production depends on how many times a day you pump on which pump you which pump you have And aaa if you're next to your baby or not, if you have a skin to skin, you have more milk. So maybe... breastfeeding should not be an outcome. I don't know. It's just something to have into consideration and maybe it's more like post menstruated discharge and or the length of stay. ...... I think breastfeeding. I presented it myself but on reflection, I think it can be challenging to have that. as an outcome.	FGD-4	PN007

Physiological/Clinical	Physiological health	Mental health in adulthood Education and professional achievement in adult life	Aaaa But also now in the references in the aaa papers that are published during the last five years, I think. Aaa It's a long-term outcome that it is very important also that has to do with social and aaa I mean, the life of the the babies as adults that they have been aaa they have been treated with family-centred care in comparison with the standard care babies that the differences in self-confidence, in success professional, success personal life, healthy personal life aaa things like that. I mean that it is not mental health in a strict way I mean they are but even in that they are less depressive or less anxious or less things like that but they are happier adults. They they really thrive if if aa they had family-centred care in the NICU. Ummmm the relationships with ummm ummm their peers in school, they umm have better uh how to say they are more better ummm they are more educated I mean they have higher education it is easier for them to be higher educated to have a they are more successful they are and also the the mother and the child is healthier, is the reaction is better during from the childhood till the adulthood.	FGD-4	PM002
Life impact	Emotional functioning/well being	Couple relationship	And also the impact in the family, the long-term impact, not only the I mean, the divorces are less, I think. I have seen papers that the divorces in these families are less. Aaa They relate them the relationship in the couple uh better I mean they because they better because they the travel, the journey to parenthood is easier is um is facilitated through this family-centred care outcomes like that social let's say some I don't know how to but to define them. But I think social outcomes and social life outcomes also are very important.	FGD-4	PM002
Life impact	Role functioning	Parental presence	And you could also determine presence. Because we know if we we know the presence differs between the southern countries and the northern countries. also, internationally. And aaa so maybe you should also ask if they were present in the NICU in the way they want it.	FGD-4	PN007
Life impact	Delivery of care	Parent-staff interaction	I also think I would ask what they felt their connection were with the clinicians. It's still connected. Or... were there were they just people taking care of my baby. So the level of interaction, I don't know how to initiate that. maybe you have.		

Core Area	Outcome Domain	Outcome	Illustrative Quotes	File	Participant Code
Life Impact	Role functioning	Parental role<br>Parental stress<br>Parental stress due to prolonged hospital stay	So I think that that our main issue is have we experienced it, that there has to be someone to manage all these things to keep the parents being able to be parents and all the noise around is regulated by the professional And so we make a decision like 20 years ago to go for case management and said, this is that so very important. The parents are so long in our hospital. They get stressed all along the way. How do we support them? I say to the parent. that is um COPE support. So you need not only sleeping feeding, resting, being nice to each other So we will help you translate and make plans for you. And that's, I think, the most important thing also to work with the NIDCAP all together and we rearrange it by the neonatal nurse practitioner That's the overview professional to help together with the parents to organize care that's my philosophy that helps that the parents have all the attention to be a parent to the child and the interaction between the parents and the child and all the other things are organized. in relationship and also give the direction for their parents, feel like parents.	FGD-5	PN001
Life impact	Emotional well-being	Parental mental health<br>Parent-infant interaction	like traditionally, I mean, we would think of I think with all these outcomes you have already yeah seen in your review but we would put them into like the category infant like infant outcomes and looking at maybe medical outcomes or well-being or parent outcomes which often would be maybe like parental mental health and but trying to aim for outcomes maybe more like Yeah. Yeah, the parenting relationship and what would that be like parent behaviours so like and linking parents to infants so like yeah interaction, co-regulation on a biological level things that uh or also and or also on a more family systems level, I think would be important to widen also from the PD003 to also what outcomes could we also see sort of in the family systems level.	FGD-5	PN002
Life impact	Emotional well-being	Parents anxiety<br>Parents depression<br>Growth<br>Breastfeeding<br>Mortality	we test the PSS NICU anxiety well with parents, depression with parents, and every core outcome like growth and breastfeeding and every complication it is um it's written down. it's very broad.	FGD-5	PN004

Life Impact	Role functioning	Parental role	we need to feel like so many people said we need to feel uh parents to be parents their parental role How can we ummmm acknowledge that and coach them in that? And I don't think we have that's already to measure	FGD-5	PN004
Life impact	Emotional well-being	Parental knowledge about infant's care<br>Parental confidence after discharge<br>Parental preparedness to discharge	I think it's like information like a bonding it's it's being a feeling like you're guided in the whole period and how do you feel that all the steps you make going home you feel like I'm well informed and didn't know what happened. aaaa I know the next steps. I aaa knew the information, what's next if I go home Who can I contact to? So I think the possibility to being a parent and also being well informed in the healthcare system. And what to do with because what are you able to know what are the possibilities to support you and your family and the child? that would be mine because I see I meet people where the the BPD, the extreme BPD I I do the I do together with uh uhh the neonatologist the follow-up and then you hear the parents telling about how difficult it is and then they of course they when they are for three, four months in the hospital they they're difficult to have the information they get a lot of information but not much information will stand So how do you help with with that? And how can we focus on what information is important and how can we give the information.	FGD-5	PN001
Life impact	Emotional well-being<br>Role functioning	Parental coping<br>Parental role<br>Parent-staff relationship	The most issue is how can we support them because they it's it's not the child who's a patient here. The parents, it feels like they're a patient as well. It's the same when you're an adult and you will have a serious disease. how do you will be solved with all the information you catch I think surprising that that for a simple like a recipe I can't produce it when I hear it for the first time. And if you hear serious issues about your health, how are you able to remember them and knows what to step and where to go and what So that's... And so central thing is how can we support these parents and can because if we can support them optimal, they feel like being parents and and get corner for me, it's the important thing because I can change society. I can change the country. I can change the rules. Of course, we can if we vote and we are to have the possibility of living in a free country. but how in who can you who how can you be free in an non-free atmosphere how can you aaaa hook that's the same thing I said about how can you be a parent if your possibility because you have your own you have your own business or you have three children at home and you have a sick mother etc So how can you still feel I'm a parent and I don't, I did the best I I I And I did.	FGD-5	PN001

Life impact	Delivery of care	Staff advocacy for infants needs	And to PN001 also indeed. Sometimes culture is used as an excuse, as a reason, as an obstacle but actually aaa we have the responsibility also to create the culture if you we used to talk about Upsola. he didn't say anything to the parents. But when the parents came into the NICU there was a bed for them. Aa You didn't need to say anything the parents knew they had to bring pyjamas. So, you can create a culture. and you want, aa as PN001 says, the parents to want to do what they actually want to do. I think one of the things that I've worked with also PN002 is familiar with my wife's work that there is a voice for the baby the best interests of the child are paramount. Health professionals should be advocates for what the baby really needs.	FGD-5	PD003
Life impact	Delivery of care	Parental advocacy<br>Separation<br>Parental involvement in their infant's care	No, no, no, no. The name's fine. I've got no problems with the name. We have enough names. We don't need new ones. hahahah But we need to implement it, which is exactly your issue and all our issues and why we're here. And in doing so in doing so to PN001's comments also and all of us Parents want to do what's best for their babies. Really, they do. It's part of their reproductive biology. aaaa when they hear and see and feel that this is consonant not with their cortical training but with their limbic feelings if you look at it in a kind of anthropological perspective. Aaa PM003 PN001 they know that this is how the parents feel. Yes, but they're obstacles, cultural things that get in the way. And so, there is a biologically based universal culture PN001. We're all the same. What is all the sameness? We have this biology of togetherness mother infant non-separation Practically, the NICU has been an obstacle for them. And so all of these names Jos have come out of a reaction to saying Parents are missing.	FGD-5	PD003
Life impact	Delivery of care	Staff education and training about FCC	regarding obstacles in Turkey on cultural wise, yeah, there may be some. Aaaa In some areas, there may be some. Maybe we cannot have mothers and fathers together. we need to share their times. But it depends. I mean, it depends on how you teach them, how you educate them. how they understand that this is beneficial for their baby. I don't see any obstacles which cannot be overruled. I mean, they could we could do it but what we have missing in here, I think the philosophy.	FGD-5	PM003

Life impact	Delivery of care	Parental empowerment<br>Parental presence	And it's also, I think about empowering parents to what to expect. I mean, also before birth is really important part of and that also obliges us to you know to to deliver that. So, what to expect? I mean, we know and that is universal. I mean, parents and babies need to be together that I mean that is the same wherever you're born in the world. And and this is what you can expect. And this is...	FGD-5	PN002
Life impact	Delivery of care	Staff attitudes	Yeah, and then the culture is is the doing that we actually, you know, what we do more, I think, in the beginning of life than what we, you know than what we see. Can we deliver it? Can we do it? And and how we show parents uh uh uh Yeah, that's... So I think the expectation is important and it also puts aaa It pushes us.	FGD-5	PN002
Life impact	Emotional functioning/well-being	Parental understanding<br>Parents coping strategies	So I think that one of the main outcomes is to think about information, giving information, dealing with information and placing information and remember information and how can we help parents to document it in an easy way that they can check and read back and and and aaaa not only to go into the file of your child but also to about the conversation you had and about all the information is given yet you can look back and because you have to deal as a parent with such a lot of things and if and it's someone helping you with information And that's why I think that case management is so very good because you are the same person who talks every week to the parents And you you will say how was last week? How are we now and what's going to happen? What are the plans for next week and we constantly looking back and going further and that helps them to keep their eyes I would say, it's the line from day one to discharge.	FGD-5	PN001
Life impact	Delivery of care	Parent -staff communication	but also, not to be afraid as a parent to ask the information like a hundred times. You have to say to parents I know you don't remember it all just please ask me. No problem. I'm pleased to tell it you a hundred times the most important thing is that ... And there is a moment when you remember it.	FGD-5	PN001
	Delivery of care	Staff attitudes	Yeah, and so that's why my main issue is the attitude of of connecting and connecting is like I don't you, you don't me, and we are completely different and we're meeting in one of the most beautiful but also frightening situation in your life. so yeah that's why I think that outdoor clinics is so very nice. You see the people after a couple of years and they're completely different parents. So, realize that in that time, they're dealing with a lot of stress. And they are different at that time.	FGD-5	PN001

Life impact	Delivery of care	Parent -staff communication	I completely agree with you. It's communication and and the timing and knowing that people are stressed. But how can we you are a perfect example of it and I think a lot of people on the ward But not everyone. How can we ummm change the culture and change the attitude on the ward. Because it's lacking there. It's lacking. It is ..People are afraid of their aa nourishing role of their doctor's role parents need to go home and go to sleep. how can we change that that that is?	FGD-5	PN004
Life impact	Delivery of care	Staff education and training about FCC	It's learning, it's teaching, it's it discussion, it's giving time for for uh And and that's also the important thing about nursing. Nursing is like looking after children or coaching parents. It's also a difficult issue because most most nurses go to paediatrician, they want to look after children. But it's a nice attitude, but it's just the wrong attitude because you don't have the child you have the parents together. So, you're more of a coaching role and teaching and learning to learn them they are coping with that situation to go home and doing it by yourself. But it's in adult care as well.	FGD-5	PN001
Life impact	Delivery of care	Staff education and training about FCC	....That's that is issue when aaa implementing family-centred care or infant family-centred care in NICU, that this can be an issue this is an issue of of roles and how nurses traditionally have been trained and that it's not equal to, aaaa you can be a very good nurse and good at doing you know all these things, but it's not equal to having the skills of teaching and guiding a parent to do the same thing. So that that is definitely an issue.	FGD-5	PN002
Life Impact	Role functioning	Parental presence SSC	So, yeah. So I think like this year going back a bit, we're talking about, I mean, what we communicate to parents, right? And we're talking about even before giving birth and then I think that's, I mean, that has to match with what we do then. So the communication and the doing has to be matching and and So all our care routines And then the outcome. So, if you're looking at The communication is that you now you're the... most important person for your child. And we're supporting you to be involved in the care and so forth. I mean, then the outcome would maybe be uh you know. Wow For one, parental presence, can we measure that? I mean, how much parents are bedside? Skin-to-skin contact the time and skins can contact so Yeah, but I think that's an important linking those the communication, the doing and and thinking about what that could Yeah, be outcome wise yeah	FGD-5	PN002

Core Area	Outcome Domain	Outcome	Illustrative Quotes	File	Participant Code
Life impact	Delivery of care	Parent-healthcare professional trust	I agree with that. (laugh) Because I think it's something you when I was thinking it's really also about developing trust. And so, I think when you're talking about the ummm challenge. Umm Well, I don't know what I'm trying to say.	FGD-6	PM005
Life impact	Delivery of care	Infants distress<br>Infant comfort	What is the baby telling us? And so, I think it's a challenge for us because It's challenging the way that we have been trained in the way that we think. you know, it's causing us to pause and really, really listen, like you said earlier, PP001, like really listen to what the family's saying, really listening to what the baby's saying and we sometimes feel like, oh, that's going to take forever, you know, because I have.	FGD-6	PM005
Life impact	Delivery of care	Individualized psychosocial support	But sometimes, I mean, from my experience. We were in the NICU. We were, umm like I said, my son was 24 weeks. We lost his twin. So, we were both a NICU family, also a bereaved family umm And So I think one of the things going into the NICU, we need families help multiple parts of their story. So, it's not just that baby that you're seeing many families have dealt with infertility, have dealt with bereavement, have dealt with a number of things going into that. So that's why it's important to look at the whole picture but I think um …. When clinicians and when everyone in the NICU really look at we are a team.	FGD-6	PM005
Delivery of care		Parent-infant trust relationship<br>Infant morbidity	We're working together to see how we can develop trust and relationships and also how we can improve outcomes because that's a really big thing. Improve motility rate, improve mobility rate umm it's… my my experience has been when we stop and do that. Umm it makes a massive world of difference in that whole experience, the whole NICU experience the outcomes of the baby, the outcomes of the family. Ummm And it doesn't take that long.	FGD-6	PM005

Life impact	Delivery of care	Holding<br>SSC<br>Infant physiological stability	Umm When the first time we were able to hold our son many weeks afterwards, I think we should have been held earlier, but that's a different story. Umm But immediately skin to skin his vitals completely normalized, completely stabilized. And umm I think sometimes we don't appreciate that, you know, because we're so focused on the numbers. that we don't realize that we don't realize that this is the way I look at it. I think the way the parents and families umm That is that baby's home and that's where they should be. They are only in the NICU because of a number of things that have, you know, kind of force them to be there. And so, I think the more we try to get them back into that safe space and at home is important.	FGD-6	PM005
Life impact	Role functioning	Parental role	So I agree with what PM005 was saying, like for your own baby, you saw the vital signs stabilized and all. So that's something that we really do see among of ... Okay, so family-centred care, although we adopt it in our centre, sometimes it is not .. the challenge is to get the buy-in from the parents as well. Maybe it's more of an Asian very traditional mindset. I'm not sure where where they expect care to be very medical driven and so that group of parents, often they take a step back and they allow the physicians to meet the care and everything you tell that is good for the baby, they will agree with it. Umm The other group of parents we have is where they adopt the, if they are involved in the FCC pretty much more normal.	FGD-6	PN009
Life impact	Delivery of care	Trusting relationship between parents and healthcare professionals<br>Shared decision making	So, we built a very trusting relationship with them. We tell them what's going on with the babies. These are the treatment options, and we get to hear their voice and concerns in terms of which approach may be aaaa better one to take. Or what are some of their concerns in terms of the treatment that the baby is going to undergo?	FGD-6	PN009

Physiological/ Clinical	Physiological health	Hemodynamic stability<br>Less respiratory support<br>Parental preparedness discharge<br>Parental stress<br>Parental anxiety<br>Communication<br>Staff burnout	So, when we observe the involvement of parents among these two groups, I mean, I don't have the data, exact data with us. But just based on experience, we can see that parents who are very involved with the care. Umm Having good relationships with the physicians, with the healthcare team as well. The babies do tend to be a little bit more stable hemodynamic, so their vital signs tend to be a bit more stable. We tend to wean them off the respiratory support faster. And eventually when it's transitioning towards home. we see that they are able to transition home pretty smoothly with minimal to no additional support required or even having a very smooth home care training, the caregiver training progress towards home. Ummm Compared to when parents are not so forthcoming to be involved very much in the NICU. we see that towards the um towards the step where the baby's starting to be prepared for home parents are usually more stressed, show a lot of signs of anxiety. And that's some of the struggles that the healthcare team do face as well. Umm In terms of what some of the challenges in terms of adopting FCC. Firstly, I'll say that among healthcare professionals, time is one of the big factor. Because as we all have mentioned earlier, FCC involves building a relationship among the healthcare members. As well as the parents and to build a relationship, it revolves around good communication, and we need time for that. So, I'm not sure how big the other settings are, but for us, we're 40 beds in NICU. So yeah, having to look through every um case. The physician team is often very overwhelmed. So having to spend dedicated amount of time for each baby, some of the challenges that we are currently facing right now.	FGD-6	PN009

Life impact	Delivery of care	Shared decision-making	So, for us here in the Philippines, family is quite broad, if you have aa Filipino friends, family includes the grandparents, the aunts, the uncles. Aaaaaa sometimes it involves friends of the family so it's quite a unique scenario in our settings in such a way that sometimes umm Even the grandparents, even the uncles, the aunts, the godfathers have a say in what to do with the patient. so sometimes when we have a family discussion. Ummm You can see around 10 relatives attending the discussion actively participating, asking questions, giving their own opinions ummm Sometimes the parents would delegate the decision making to the grandparents and our healthcare setting is also quite unique in which there are uhummm Actually, I'm a physician in both a private hospital and a government hospital so those two hospitals have a unique setting in which private hospitals tend to tend to talk more tend to gather more to the whole family. We're in the government settings in which there are tons of patients and we could only allow specific times to them Um. the family-centred decision-making is not that um specific or not that detailed ummm so that's my experience here in our country. Thank you	FGD-6	PD005
Life impact	Role functioning	Parental involvement<br>Parental role	May I add some more on challenges of family-centred care? I'm very I agree a lot with PM005 She said that she said that....umm Parents must be involved. Parents must be listened, and parents must be ummm let in the NICU and stay in the NICU. And the first challenge for the professionals I think is is having an approach that help the parent to be the most important person for the baby, not visitors and in Italy. We are still struggling with open the NICU 24 hours, seven days a week. And this is the first step to implement family-centred care. So, this is one of the main challenges, I think for healthcare professionals.	FGD-6	PP001

Life impact	Delivery of care	Restricted parental involvement<br>Staff resistance<br>Parental presence<br>Infection	Okay, so for us in the Philippines, family-centred care, the challenges that we could see what the structure or the facilities. Because most of our facilities don't have rooms that may accommodate the entire family uh to be with the parents. But most of my hospitals that I'm affiliated with, we allow them to visit any time of the day, even at night. Unless there are some procedures that involves the other babies so uh, we usually ask them to leave for a while. while there are some ongoing procedures to the other babies can find the need. We don't have any private rooms per patients.... So sometimes it's a challenge for us to maintain or to keep...aaaa to maintain privacy for each individual patient.so but most of our clinicians are open to to family-centred care in which they allow all the other family members sometimes even extended time to visit the NICU. But umm there are some who are still resistant to that thinking ummm and they are more concerned about the infection control. when you have a lot of visitors or a lot of family members going in and out of the NICU. So, for them, that might introduce infection to the other patient thank you.	FGD-6	PD005
Life Impact	Delivery of care	Staff attitudes towards FCC	May I add some more on challenges of family-centred care? I'm very I agree a lot with PM005 She said that she said that....umm Parents must be involved. Parents must be listened, and parents must be ummm let in the NICU and stay in the NICU. And the first challenge for the professionals I think is is having an approach that help the parent to be the most important person for the baby, not visitors and in Italy. We are still struggling with open the NICU 24 hours, seven days a week. And this is the first step to implement family-centred care. So, this is one of the main challenges, I think for healthcare professionals. And the big challenge is to change the mind and the approach of the professional because intensive care unit is intensive care And as PM005 said, family-centred care should be transversal, you said Is it correct in English? So, all the intervention in the NICU should be guided by the principles of family-centred care. We cannot divide intensive care and family-centred care. They are one approach. ... I think this is my experience	FGD-6	PP001

Life impact	Social functioning	Parent support from family members	I'd just like to add that my setting is also pretty similar to PD005 So for infection control purposes, we only allow parents and grandparents and it's only two visitors at the bedside at a time. So, in that sense, I understand from some parents that umm Maybe the grandparents are no longer around and maybe their social support is like the mom's sibling or the dad siblings. But because of regulations institutions regulations, we don't allow such visitors. So, I feel that that can actually affect the support that the parent receives while having a baby in the NICU. Yeah, and otherwise the late as for the environment layout, we don't have private rooms as well. So, it's typically four babies to a room. We currently don't have a rest area or a room with a couch for parents to rest so that this allows parents to stay for long hours within the setting the ICU itself yeah	FGD-6	PN009
Life Impact	Delivery of care	Staff engagement	I've actually never received any negative emotions or feedback or even comments from them. So actually, that meant me to realize that parents do want to be a part of the care of their babies. It's just a lot of fear and stress that they have and for a start, it's very important for us as healthcare professionals to hold their hand to guide them through the initial steps of getting to know the baby, born with the baby and actually that's what a lot of parents want but it's just a lot of fear that was stopping them.	FGD-6	PN009
Life impact	Role functioning	Parental shock<br>Parental role	when you know the NICU itself is a very traumatic space and any of us any of us, if we experience something that just flips our world. You do. You're right. You do have those feelings of shock. And I think the um and fear. umm But I think it's something thinking about remembering that umm the natural way of parenting has been interrupted when you go into the NICU. So, you sort of almost lose those natural parenting feel because you don't know. You know, you're sort of in a bit of a state of shock.	FGD-6	PM005

Life Impact	Role functioning	Parental role	Umm But I think the educating bit, because you are right. We have I do a lot of work with parents in different umm realms and And there is this, there are parents who feel like, well, I don't really know what to do. So, I can't do so they they may be stepped back. Umm Also believing that as the clinicians, you have all the answers. And I think that's, again. The way we're talking about earlier about how do we shift the mindset that we need to change that and say that we all have answers.	FGD-6	PM005
Life Impact	Delivery of care	Staff attitudes towards FCC<br>Parent-staff communication	The way we're talking about earlier about how do we shift the mindset that we need to change that and say that we all have answers. That you know the clinicians have answers. Also, the parents have asked the babies have answers so I think It's changing the way that we do, changing the way that we care And remembering that even our language, and I'm not I know that, I mean, everyone on this call is, you know, is very passionate and intentional about what they're doing but Even the word we say visitors I think we should throw that out (Hahaha). Because parents aren't visitors. You know, you shouldn't be a visitor to your own child umm but again, that's a part of our that's a part of the medical picture. you know that we've created. And I think umm it’s one of the challenges is chipping away at that and saying that You know, even the way that we talk about parents and talk about clinicians. It's not us and them scenario. It should very much we’re all on the same team. we're all working together. We're all coming in at different parts of the picture.	FGD-6	PM005
Life Impact	Delivery of care	Parent-staff communication<br>Shared decision-making<br>Parent-staff trust	My son is now on He had severe ROP. So, he lost a lot of his vision. So, he's legally blind now. But I found that that when i found that when the clinicians that actually sat with me and we didn't sit for 20 minutes. The clinicians are that on a daily basis, you know, one minute, two minutes three you know those kinds of things umm those are the clinicians that we have to this day, 18 years later, still have a relationship with. And so, I think it's remembering that it sounds very like Wooby woobie, but it's really about being human. And that we are connecting with each other. We're connecting with parents. So, things like, you know, what did you see? asking those questions when the clinicians were asking us, what did you see? What did you notice? How did you feel? Those were very, very, very, very important ways of developing relationship and it didn't take five	FGD-6	PM005

			minutes. It took those daily one to two minutes where you just felt a connection with the clinician. And I've been on the other end where um care was rushed. You know, it was like, I got to go in, I have to have to see all these babies and I need to and we were very much not considered and ummm really broke down my belief in whether that that particular clinician cared about my child or cared about us and so I mean, having both ends, I think it's very important to um to ummm recognize that the education has to occur not just for the clinicians, but also for families. Ummm And recognizing that we are kind of all in it together just to wait, we just need to change the way that we think about health care.		
Life Impact	Delivery of care	Staff educating and training about FCC	I would like to add that a umm big challenge for the institution and for the yes for the situation is to promote a relationship based care instead of a task oriented care, which is care in a rush one patient after the other without even knowing if it's a girl or a boy or who the parents are. And I think that this change implies a lot of education also on soft skill communication and interaction skill and active listening because The professionals of the NICU are super skilled in high specialized topics. But no education, at least in Italy, is dedicated to communication, interaction, listening and whatever is not technical. So this for me is super important as for the institution to promote this kind of education for the NICU professional.	FGD-6	PP001
Life Impact	Delivery of care	Parental competence<br>Parental preparedness to discharge	I think they are evidence-based and ummm I can tell you our experience because recently we ummm collected opinions from parents who had a very preterm baby in our unit. We are a NIDCAP training centre. So we use family-centred care and NICU-based care and we interviewed parents asking them how was their path from admission to discharge and the fact that the care was based on family centred care and the NIDCAP observation helped them a lot to develop parental skills how to be parents, how to get involved and they benefit a lot from this type of care because also the interaction between them and their baby and them and the professionals, the NICU professional was very um ummm How can I say this in English? was very easy. They felt involved in the care they felt listened. And they felt ready to go home. I don't know if it was clear.	FGD-6	PP001

Life impact	Emotional function/wellbeing	Staff burnout	I was just going to say when she was talking, I think she raised a really good point. And also, before like she was talking about the being overwhelmed with... Just things you have to do. I think it's important with family-centred care because we're talking about kind of having the parents centre, but When we're talking about a collaborative effort, I think we cannot forget the needs of the providers, the needs of the ummm clinicians and professionals and realizing that Just in my experience, they've also expressed that they're fearful and overwhelmed.	FGD-6	PM005
Life Impact	Delivery of care	Parent-staff communication<br>Parental trust<br>Shared-decision-making	there's a lot of pressure that is put on everyone within that space. And there's a lot of ummm trauma that's experienced, not just by the families, but also by the clinicians. So, I think that goes back to when PP001was talking about that relationship based we have to look at all the relationships that are happening. And I think it's something about How do you do that? in a way to where people don't feel like it's something extra that it's not something that's added on to what they're already doing. You know, we have to look at it in a way to where this is the way we do things and it becomes an integral part of you know ummm our care, the clinicians that sat with me for one minute to say this is what we're thinking also to say You know, I'm not quite sure what outcome will be This is what we're thinking umm but And that, for me being able to say you don't know was also very human. And also, very powerful. Ummm Because it's like, okay. there could be multiple things that happens umm and and we can make a decision to get as family, we can make a decision as much as we possibly can with some kind of informed decision. But knowing that there wasn't a guarantee for me, that was quite ummmm It was more real. It was a much more real response versus ummmm a how do I say? standard response ummm thinking that clinicians know all the answers. And when the clinicians would say, you know what, this is what we know, this is what we've experienced. I'm not sure if it will work, but this is what we experienced to me, that was much more real And that helped to develop the relationship because I knew that that clinician was going to be honest with me regardless of the outcome. I don't know if that makes sense when I'm saying.	FGD-6	PM005

Life Impact	Delivery of care	Shared decision making<br>NEC	I do remember one in particular um when ummm his main neonatologist was thinking that he was beginning to show the signs of NEC. So necrotizing enterocolitis. Ummm And the gastro team was saying he wasn't, so he was getting sicker by the day. I mean, but I mean, sorry, by the minute, really and there was one team that was listing and one that wasn't, and we get into this weird battle (haha) of, you know, and I'm in the middle of something has to be done because my son is deteriorating fast. you know um Eventually, and so the the team their reasoning was we don't know for sure if it's NEC. So why do we want to put this baby on You know, for emergency surgery and he doesn't make it. That was their kind of reasoning. But on the other side, the neonatologist was the one who had a relationship with, was saying, well, I think we need to look at it a different way. So basically, there was a medical decision that had to be made but the way that it was being made was very different. One clinician was looking at a whole picture of the numbers of, the baby, the family, what's happening, and another team was looking straight just at the numbers. End of the story was he had to have emergency surgery. He did have NEC. it just what it had it had to be you know um you know, some of this testing had to be removed. He had to be resection later.	FGD-6	PM005
Life impact	Role functioning	Mortality<br>Shared decision-making<br>Parental role<br>Parental involvement	umm family success aaa One thing is for the baby to survive. But another part would be how You involve the parents in the decision making just in case the the baby doesn't survive, it's important that you make them feel umm as a part of the team who made them feel that they are parents for that limited amount of time that they had with their babies. So I think that's also important outcome in which they are able to be parents, no matter how how short it is or they are able to at least somehow take care or be involved in taking care of their child.	FGD-6	PD005

Life impact	Role functioning	Parental role<br>Bonding	I think that If you're looking at like the success rate of the family-centred care in the NICU, looking at parent outcomes, one of the things will be how much of a parent does the parent feel to their own baby like ummm the enhanced bonding that their parental involvement actually allows whether they feel close enough to their baby because of the activities that they could do, like maybe the tube feedings, diaper changings or maybe even adjusting some of the saturation probes, things like that.	FGD-6	PN009
	Delivery of care	Trust relationship between healthcare team and parents	ummm and one other aspect would be how much effort trusting relationship they feel that they have with the healthcare team. And that's not just with the medical team But the allied health professionals as well because It's a whole team that is actually managing a premature baby in the NICU	FGD-6	PN009
Physiological/Clinical	Physiological health	Neurodevelopmental<br>SSC<br>Infant distress	Other outcomes for infant outcomes, I think one of the things that we often talk about will be the neurodevelopmental outcomes. Even when we encourage parents to do things like skin-to-skin kangaroo care ummm This is one of the aspects that we always say is, you know, it helps to stabilize the baby, keeps them calm. And eventually the long-term thing we're going to look at is improved neurodevelopmental outcomes. So, I feel that towards discharge or even a post-discharge evaluation or measurement of infant outcomes, neurodevelopment would be one of the key aspects to look at.	FGD-6	PN009
		Ventilator support<br>Oxygen therapy<br>Increase from tube feeding to oral feeding	as well as how well the baby is able to perhaps, we know ventilatory or oxygen therapy and even more from tube feedings to total oral feeding. So, we often counsel or rather share with parents that being more involved in the care, participative in the care will help their babies to progress well and faster in these aspects. So, these are some of the outcomes that I feel that are would be worth looking at in terms of outcome measures.	FGD-6	PN009

Life impact	Delivery of care	Parent-staff communication<br>Parents trust relationship	Ummm As for staff outcomes. So for the whole healthcare team, firstly, again, looking at the communication and the trusting relationship that they feel they have with the the patient and the family that they're caring for would be important.	FGD-6	PN009
	Emotional function/wellbeing	Staff stress	But another thing that we also do need to look at would be the stress levels that the healthcare team feels umm Given that having a Because now they're not only focusing on the baby in the centre of the care, but the family as well.	FGD-6	PN009
Life impact	Emotional function/wellbeing	Parental financial support<br>Parents emotional support from staff	And so, some people may feel that having to juggle with multiple aspects of the infant and family care may lead to higher stress levels Because parents are stressed as well. Parents may require further social support and other supports like even financial constraints that the whole healthcare team would then need to look at. So, this can actually lead to a lot of emotional or even mental stress to the healthcare team up field yeah.		
	Emotional function/wellbeing	Staff stress	Sometimes it leads to stress to the staff in which parents are somewhat more demanding or more anxious umm the first few times that they are the one taking care of the babies. So they would often call the nurses asking them to check umm If they are doing okay and asking them if what they are doing It's good for the baby..	FGD-6	PD005
Physiological/Clinical	Physiological health	Neurodevelopmental<br>Infant's physiological stability<br>Growth	Umm when it comes to the implement, we also agree that the neurodevelopmental outcome in which we see the babies more stable or grows faster. ummm they could be easily win if the parents are more active in taking care of the babies	FGD-6	PD005

Life impact	Role functioning	Parental presence	umm in the government hospitals in which in where there are multiple sometimes dozens of babies needing intensive care and the space doesn't really permit sometimes we just only allow the parents to see their babies for a limited time to allow or to accommodate other parents to also see their babies in which some we lose some of the time that we let them umm take care of their kids. Thank you.	FGD-6	PD005
Physiological/ Clinical	Physiological health	Neurodevelopment	I think that is really key. Because for me, one of the outcomes is not just the outcomes in the NICU. But what happens after NICU? But what we do or don't do in the NICU has a significant impact on what happens so like with sensory development you know um if if we're if we're having alarms going off all the time where people, you know, that is really affecting the sensory development, neurodevelopment of that baby.	FGD-6	PM005
Physiological/ Clinical	Physiological health	Behavioural	we're even seeing now there's studies now that’s showing um behaviour and developmental issues 18, 20 years post NICU Because of something that hasn't been done or done or hasn't been done. So, I think it's really important when we're talking about outcomes that we're looking at we don't forget that developmental part.	FGD-6	PM005
Life impact	Delivery of care	Staff communication to cope with different background of parents	For us, we umm Yeah, patients of different races and naturally they have different cultural backgrounds and beliefs expectations as well in the way they believe how the baby should be, how care should be delivered I think what what really helps is that during the early days of admission, they often get multiple a little short sessions of updates from the physicians to to sort of set an expectation of what it's going to be like from here. We may not know what's the final outcome, but this is what we're going to do for the baby, and this is what we're going to do to support you through this NICU journey. And then moving on from there, the different touch points is what makes it important.	FGD-6	PN009

Life impact	Role functioning	Parental involvement	some of the cultural practices here would be to have a period of confinement where the mom is not allowed to leave home for a month to up to six weeks, depending on which culture and tradition they follow so they're not able to umm come to the hospital to visit the baby. So usually in the early few weeks, it's always the dads that come and visit. And sometimes because the dad's paternity leave ends early, they're not able to take the full lease …so beyond a week or two, they're back to work, they're visiting hours are rather the parent visitation to the NICU is much shorter. Unfortunately, I guess that is just the way the system works here as well in terms of our maternity leave and paternity leaves are not too long, they're just for about four months now for moms. So, what most parents do is that they just take a little bit of rest they go back to work to to save the last bit of leave for when their baby barely goes home with them to help them spend more time at home with their baby. This is one of the things we feel that kind of limits aa their interaction and involvement with the baby's care. But otherwise, in terms of cultural differences in the way that we are allowed to do certain care or not allowed to do certain care. That is not something that is not something that is really a problem for us over here. Yeah.	FGD-6	PN009

Life impact	Delivery of care	Staff cultural competence	I think when you're talking about cultural differences, we have to be incredibly sensitive to what is it to those many things that could show up for families in that way. And we have to be open to adjusting maybe the way that we provide care. And I was sort of thinking. You know, when I was hearing you speak one very simple way, and this is, I've seen this happen in different NICU units is you know when you first, when the baby's first there, you know, say, what is important for you how do you want us to How do you want this relationship to be and just A couple of queries like that is very important because you'll get immediately you know um umm what's important for them culturally? And what's important for them, you know, it makes me think about a friend of mine who's Jewish and they lost a lost one baby and the baby in the NICU. And part of their culture of bereavement was umm they don't You know, they don't wash, they put in, they wear black, they put cloth, and they rip their clothing as a side of mourning. Ummm So, they were, long story short, they were referred to social services and refer to the authorities. Because the hospital was thinking that was thinking all kinds of things (Hahaha), you know, but not understanding.	FGD-6	PM005
Life impact	Role functioning	Bonding	I think that's and for me, that's also a part of infant and family centred care that you're actually looking at what is important for that family and… helping them through that. You know, though, you know, is it important to touch? You know, is it important to to change the nappy, change the diapers, those kinds of things. And I think it's, I think all those things are a part of care and all those things we have to take. We don't we shouldn't take those for granted and think that they're insignificant because they are You know, for me being able to change my child's nappy, being able to touch my child because the incubator is an automatic separation. And the fact that you for a few days you could only put your little pinky finger in. That does a lot. That has a lot to do with bonding has an adverse effect on bonding	FGD-6	PM005

Life impact	Role functioning	KMC	Actually, the majority I think would be the Muslim ummm the Muslim...people here in which they are more conservative we could not really do the kangaroo mother care they are more...mindful of their surroundings with the Muslim, they don't want really to remove any part of the clothing so we need to provide them with the really private environment in order for them to do their KMC, in order for them to do their breastfeeding. So I think that one... aspect aaaaa with regards to the culture or the religion how we approach or how we try to accommodate their beliefs.	FGD-6	PD005
Life impact	Role functioning	Parental knowledge infants care and treatment Communication	But often times he always said, and this is you know, English isn't his first language ummm and so because of that, you can understand things in a slightly different way so and you're thinking you're you're thinking you're thinking that, but it's not the same. And so, I think, um. I think it's important to pause sometimes and think about What are we actually asking families? How are we asking them? The timing is so important. you know experience we were we received a lot of information on the front end, and it was too much. Because all we were worried about was, we already lost one child, we're losing the second one. So, I can't process all this information. So, part of that is about when do you provide information to families. umm Do you provide all of it or do you provide just little snippets? And you won't know that unless you're stopping and ask, you know, we do have things to share when, you know, when do you want it shared? How do you feel? Do you need to have a moment ummm We can come back tomorrow. Who says we have to get everything in 24 hours? So, it's changing the way that we you know, do things. And I think being very sensitive, I mean, the fact that you even as a hospital in the Philippines being sensitive to oh we may need to look at a different way of supporting these groups of I think it's wonderful......We don't have that a lot sometimes (Haahaha) in the You know, some of the hospitals here, I think sometimes.	FGD-6	PM005

Core Area	Outcome Domain	Outcome	Illustrative Quotes	File	Participant Code
Life impact	Role functioning	Parental involvement	Two family. Two ..Two parents maybe father and mother or grandmother come into our ward and take care of the baby maybe in our ward because we have no room for.... Then they built this building. they didn't design the family centre room for parents in our ward. So, parents in our ward so um in our ward the parents just taking ....umm the parents just taking For daytime. And at nighttime they made back to home. And the next day they may come again. And at this time the The family may take care of the baby, but this baby may have some..iiii have something the nurse should teach them.	FGD-7	PN011
		KMC	Yeah, because our unit is all for the very preterm babies. So... At first, we will let the parents to come in to visit their babies. And aaa after the baby was transferred to the iiiii For example, if the baby is not very severe we will let the baby... parents to do the kangaroo care. Or do the... Or do the iiiiii daily life care like change the diaper or do the...iiii do the... I'll just check temperature like that. And then the parents can participate in the iiiii take care of the babies. And after iii that we also need to teach the parents how to do the care of the babies. ummm If the baby is stable, we will let the parents come in. And they can stay ummmm with the baby for 24 hours. As they like. And... Yeah, we will do the I think we will do the family centre care in our ward. But just as PN011 said, we do not have so much space. And you know, in our umm we have about umm 45 45 babies every day. 45 and umm in one room we may have 12 ..12 babies. and.......So we comfort both suffer like that for the parents can come in to do the kangaroo care. So, in our unit, the parents, if they want to come in they need to to take a queen. I'm not very sure how to say it if today we just have five parents can come in and tomorrow, maybe hmm we have four parents can come in and the other babies their parents cannot come in at the same time. Because we do not have so much space for all the parents to come in at the same time. So we have one nurse to hmmm arrange this visit and arrange this hmmm family-centred care. Yeah.	FGD-7	PN012

Life Impact	Delivery of care	Staff workload	we think the family-centred care from our nurse view, we think it's very good. At first, ummm when we want the parents to come in. As a head nurse, I think maybe the nurse the the bad scenario will not like it. But now they are now like it because the the parents come in and they can come forward to the babies. And the nurse think they have...umm their workload is their workload is lower.	FGD-7	PN012
Life impact	Role functioning	Parental presence	But at first, they would not have so at first we think we will have so much parents they want to come in but now we think. Now we found it's not like that because in China you know the the mother needs to stay at home. For one month. For that one month, they do not go out they do not come to the hospital. So we do not have so much parents want to come in. hiiiii And we also have, but the parents, if they want to come in, they stay in our NICU, they will feel very good.	FGD-7	PN012
Life Impact	Delivery of care	Infection	Yeah, pandemic. Yeah. So, if just one one one week ago. We have when parents come in and after they leave the baby have the flu. So, we think the parents hmmmm bring the broad the flu in our NICU.	FGD-7	PN012
Life impact	Role functioning	Parental knowledge infant's care and treatment Parental preparedness to discharge	Because there are so many. Because the parents didn't come into our ward when the baby come into our ward and sometimes when the baby is stable, but they may take tube iii feeding our ventilator back to home. We ask the parents if you want to ... if you want to come in our ward and we teach you and then you can take the baby home and then the parents come into our ward start family centred care maybe needed two or three days. At this time, the nurse was very busy because the we have many things showed to teach the parents how to take care of the baby, how to tube feeding, how to use the ventilator and how to iiiii evaluate the babies... condition So, the parents need two or three days to learn these things and learn these things maybe the the .... three day the first day they will go back to home and then another family will come in and start. So, the nurse was working so many work to do every day......And there's sometimes oh we'll meet the parents the father didn't want to work. All the things mothers do these things and then the father just see. And then he say, oh, no, no, no, you do not right you should You should do something like this it is but they just sit and don't do sometimes we're sure to uh we will make ummm some like the father take in and take in...	FGD-7	PN011

Life Impact	Emotional function/wellbeing	Satisfaction of parents<br>Parental anxiety<br>Parental stress<br>Staff workload<br>Parents-staff communication	Yeah, I think firstly, we need to to evaluate the satisfaction of the family and also Maybe the iiii anxiety and of the parents. hmm Or the depression of the parents. Hmmm And we also need to say hmmmm there is... Yes, or not the nurse hmmm nurse the workload has a lower than before. And also, hmmm the nurse hmm receive the family-centred care or not. And also, after the family centre care the implemented of the family-centred care may be the ummm the ability to to to communicate with the parents of the nurse is increased or not.	FGD-7	PN0012
Resource use	Hospital environment and resource use	LOS<br>Infection	And we also need to say the parents do the family-centred care shorten the hospital stay is whether or not And also we need to tell us the family-centred care decrease or increase the the infection or not.	FGD-7	PN0012
Life Impact	Delivery of care	Parents financial constrains<br>Doctors perspective about FCC	Yeah. And ummm I also want to know the economical economical umm aspects but I'm not very sure how to evaluate the when the parents come in, maybe the cost or the from the economical uh aspect if the family-centred care have some beneficial, but I'm not very sure how to evaluate it. and we also need to not only the nurse we also need to know what the doctors think about the familycentred care and do they think it's a very good thing or are not and we need to evaluate to the doctors and ummmm maybe you.	FGD-7	PN0012
Resource use	Hospital environment and resource use	Readmission	Yeah yeah readmission in our ward the baby coming to our ward because different ummmm kinds of reasons for inflation. Of the parents of the parents take not so good at home they were all inflation again and come back so this is very important to evaluate the ummmm family centred care intervention.	FGD-7	PN011
Life Impact	Delivery of care	Parental trust healthcare professional	Two hours and we will let umm mailing courses together not to just not just one teacher we may have three teacher and maybe one is talking about the the breastfeeding and another made talk about the NRP and maybe the third one we'll talk about the hmmmm I'm not very sure maybe talk about how to recognize the condition of the infants at home. Yeah, yes, yeah. Yeah, because we think that class is very good because at first you teach themes, they will trust you. They will trust you it's very good to build the trust. Yeah, so we think that is very important. Yeah.	FGD-7	PN0012

		Parental trust healthcare professional	Yeah, you know, because hmm in our NICU, because not every parent every day can see their their baby. So if we teach them at first and they will know the condition of their baby and we they will also know what we have done for their babies. And... ummm ummm if way we'll do some make some decision and we tell the parents they will trust me they will say yes but if they do not trust you they will think their baby do not get the good hmmm medication or care in our NICU because they do not can see their babies every day every minutes.	FGD-7	PN0012

Core Area	Outcome Domain	Outcome	Illustrative Quotes	File	Particip ant Code
Life Impact	Role functioning	Parental presence	Yeah. Now, PM007 says something which is profound PM007 says said it in a very definitely straightway said that it is zero separation. Do we have that? Does our system, the question will be, do we have facilities that will encourage zero separation we are getting there.	FGD-8	PM006
Life Impact	Delivery of care	Staff education and training FCC	she can reassure us that even in Europe. We do not have family-centred care in every NICUs. And what and still, umm I can see with the professionals that we are struggling what family care centre means what do what it means to them, what it means to the doctor, what it means to the nurses, what it means to the even for the cleaners and what it means for the parents. It's completely different things, a completely different approach. And most importantly, it's not only a question of structure but education. Also, because I visited already NICUs where they are not separating the babies at all I mean, I've been in prison for a triplet 26-weeker in Brussels where they've been putting in on the chest of the mother immediately after birth and they've been stabilized in the mother and the father after the birth and then from that moment. The mother and the father was with the triplets throughout the three months they were there And then... umm I've been in an NICU in France where they think that they're doing family centre care but it's obvious that It's only when the mother is getting better can start to visit the baby and they call them 20 family centre care because they are open 24/7 When I asked the mom, are you going during the night also? Because she has the right to stay ummm at the hospital and she's saying that Well, you know, the nurses during the night they don't want to see me there. I have the right to go in, but they are not so friendly and welcoming. So, when we are speaking about family centred care, we have to somehow precise the definition what we call family centred care.	FGD-7	PM007

Life Impact	Delivery of care	Shared decision-making<br>Parental competence in caregiving	we have to speak about religion also because we can see that with different religions with different type of culture not as there were moments when you cannot even speak to the mother because it's the father or it's you have gypsy families. I'm sorry to say that the the traveller, we call them travellers. The grandmother is the boss of of the family. So, you cannot even put up a question to the mom. Because she cannot make any decision about, not even about her her baby because it's the grandmother who is giving her or making the decisions. So it's somehow in our education as the healthcare professionals and the families also because we have to educate them throughout the whole NICU stay to become competent parents. As PM006 said, after going home and then going through umm aaa the follow up.It has to be it has to be in the education system also And as I said, not only for the professionals but for us parents also to understand not only written and unwritten rules of NICUs and cultures but But umm we are people at the end so and we are coming from different backgrounds from different culture and from different religions and we have to somehow respect it. But as we say, what is written as family-centred care as a protocol or written or guideline and what is happening in real life somehow, and most of the cases, it's completely different Unfortunately.	FGD-7	PM007
Life Impact	Role functioning	Parental presence	Because... you can't explain what family-centred care for me. It looks like when, as a mother I do not have... the space to be ... I do not have nest my baby if I choose to stay in the facility or the kind of space and the environment that will encourage other key close members of the family to be part of the care journey if I have to stay there with the baby.	FGD-8	PM006
Life Impact	Emotional function/wellbeing	Staff well-being<br>Staff education and training	I understand. But for me, when we are speaking about family-centred care for me it's equally important the state of the nurses that how they are physically, mentally, number of the nurses and education of the nurses. Because if you are having missing stuff, if you're having like what we have huge problem, for example, in this moment, Hungary. Low number of staff and low educated staff It's a huge burden so we cannot call it family-centred care because when we are looking for the components.	FGD-8	PM007

Life Impact	Delivery of care	Parent-staff communication<br>Staff shortage<br>Family unity	I had um discussion between you know you know renee flaking a Swedish nurse I mean we are looking for the sweets as like they are the examples of family centre care and so on And she's keep telling me that, you know, somehow as she is an IBCLC, she was thinking that the more quickly they will become family-centred care and so on ummm having the breastfeeding and the mother's own milk, the the amount of mother's own milk. It's going to be getting higher and then they're having when they will have the private rooms and then so on, it's going to be heaven and then finally, it's decreasing because Everyone is thinking that the moms are doing great in the private rooms. But as because they have no time to speak with them, no time to take on them, no time to check on how they are just pumping or what they are doing they are so isolated. So, the amount of mother's own milk just decreased in the past few years and they have less and less umm ummu Breast milk in the time of discharge. So, ummm we are thinking that yeah, we are doing family-centred care for different reason and for more kangaroo care for more breast milk. That means that the outcome of the child is better and more attachment and so on. But they have the opposite ummm experiences. Just because they do not have enough nursing. Just because they have not enough time to take care of their job while they are there, and they cannot be helpful enough for the parents and for the mothers….Plus, for successful breastfeed, you need a father.	FGD-8	PM007
Life Impact	Emotional function/wellbeing	Staff emotional support through communication<br>Parental guilt<br>Parent feel alone	I remember… I remember when I was close to the incubator, and I preferred that there was only two of them. They were not speaking a lot. But they saw me when I was desperate. And they just came to close to me. They just put their hands on my shoulder, and they just stayed with me for a few minutes. It was more helpful for me And I'm keeping those memories more kind than those ones who try to explain to me that why I don't have to cry because if I'm crying that the baby is going to feel it and then um and then it's going to drop the saturation and so on. So, I have to leave So, um. When we're speaking about mental health. aaa It's of course it's communication and and sometimes it's communication and sometimes You don't need words. You just need a gesture. Just a touch or just a touch just to feel that you're not alone because on that moment when you're there you are feeling extremely alone.	FGD-8	PM007
Life Impact	Delivery of care	Staff education and training towards FCC	I highly advocated that the nurses, the healthcare of professionals aaa I mean, family centred care program should include training conscious continuous	FGD-8	PM006

			training of healthcare professionals aaa to change attitude for attitudinal change towards care delivery. And, you know, that would be interesting		
Life Impact	Delivery of care	Staff cultural competence	in Europe, as we were speaking about culture I mean when we it's the travellers is just one thing but for example when you have a Jewish mom in front of you on Saturday. Aaa She cannot bump because in Saturday, she cannot do any work And you have to do every work. It's something that you have to know immediately or instantly by yourself that, okay, Saturday morning at midnight uh well Saturday night at midnight from that moment in every two or three hours I have to go into her room, and I have to pump her because she's not going to pump because she cannot do it. So it's just those things that um it's coming with ummm with education as we were speaking about it but when we speak about education and communication and and beliefs and misbeliefs misbeliefs are so important as you said	FGD-8	PM007
		Parental knowledge infant's care and treatment	But in evidence-based misbeliefs like for example what I can run out of the words when they are putting the milk, the bottle of the milk in the hands of the mom on saying that okay ummm it's better than breastfeeding because then the baby is getting weight quicker and then you can go home earlier. It's not true. It's not evidence based but this is what they keep telling the mother. So, at the end They do not want to breastfeed because they want the best for the babies so gain read as quick as possible and go home. So somehow, they are giving this bad aa information to the parents and other thing which is also makes me crazy and it's not only in my country a lot of European countries that on level two NICUs, but the babies are also always on on the aaaa the position when they're on the belly on the belly position so they are in in tummy aaa and they are sleeping on tommy. And then the babies are going home like that. But they are not teaching even the baby, but the parents that you have to put the baby on the back because it's safer for your child. And when we are telling this when they're going home after discharge and we are telling this to the parents that it's not safe that your baby is on the belly they're answering, but this is how they were sleeping at the NICU also. And then I always have to repeat to them that, yeah, but in the NICU, you had all of the alarm system, and you had the doctor and doctors and nurses so if there is something going wrong, they can help your baby. But when you're at home alone. How quickly the ambulance can arrive. If the ... For example, the alarm is going to set on So it's those things when we are speaking about not only	FGD-8	PM007

			education on communication, but education on evidence-based updated evidence based umm care so it's it's uh.		
Physiological/Clinical	Physiological health	Neurodevelopmental<br>Infant pain<br>Infant discomfort<br>Feeding	Well, you know, when a child having a severe ummm problem to grow up umm Of course, things can happen around birth or before birth. But things are happening around after birth. And it's because of the often..... that we are not taking consideration of the neurodevelopmental care of the baby. Of the neurodevelopmental ummmmm that how they're growing is just simply not considered. They are in noise, they're having pain. As I already started to speak. So how they are eating, what type of feeding problems and eating problems they will have problems in the next few years and so on. Everything is reflecting on family-centred care. So that's why for me it's short- and long-term outcome of the baby.	FGD-8	PM007
Life Impact	Emotional function/wellbeing	Couple divorce<br>Parents coping	, they did some kind of studies around that but it's only qualitative research around me. That most of these most of these marriages are breaking up because they cannot deal especially in long term with the trauma that with what they faced around around birth. And... And that's because they are not having not only couple counseling but mental health counseling or or family counseling um these maybe something else is triggering the divorce or the breakup but they are not staying together.	FGD-8	PM007
Life Impact	Emotional function/wellbeing	Mothers' well-being	What components of of the of the the design, family centre care design is targeted at the woman's mental well-being, or you know care during the stay there.	FGD-8	PM006
Physiological/Clinical	Physiological health	Infant survival<br>Infant morbidity	So, then I will agree with when it comes to baby the next five years. What the baby's health looks like what the complications look like, what's the Uh... You know. Whether they are surviving or they are not, whether we are experiencing a certain stage Of a medical condition as a result over a certain group of babies.	FGD-8	PM006
Life Impact	Role functioning	Parental presence<br>SSC<br>Infant feeding<br>Infant sleep	What I was thinking that what maybe we can use as components. Aaaa We have really nice studies which been done in the past five, six years regarding that. And there is one in mind. It's Rui and Jacques Cezanne who did that or about family-centred care and components. And they aaa defined eight components which has a huge impact on short and long term of the baby and the life of the family. The first one is access to the baby. Second is... ummm aaa psychological help. First is skin-toskin, zero separation, skin-to-skin contact, fourth is feeding, fifth is um protecting sleep protect sleep, aaaa and then we have... to the environment	FGD-8	PM007

			And then we have another two. But I can’t remember (Hahahaha) But I can send it to you uh		
Physiological/Clinical	Physiological health	Scoliosis in teenager<br>ROP	we are doing the follow-up and then uh aaa we always keep forget about that, that these kids becoming teenagers in one moment when they have huge hormonal problems and if they have been damaged somehow they have problems with uh with problems with learning or they have physical aaa handicap and so on. In this age, it can make it worse they can have secondary handicaps like for example my son had I never thought that that is going to happen that a simple scoliosis, which is somehow something normal for every teenage child or youngster aaaa and when their limbs are just growing and they are starting to moving so differently and then they having balancing problems. But for physically handicapped child, scoliosis can be a secondary handicap And then it's causing another problem because it's causing are we going to operate doing the surgery for that or not how it's going to ummm infect the life of the child and then for the family and the parents or the siblings and then so on so it's somehow, I kind of feel or ROP. When the ROP is getting worse at the age of of teenage years. Ummm Because they're growing, and the retina is becoming detaching and then so on so ummmm I would say that the outcome somehow of the child has to be defined when they're starting the adult life. That what type of adult life they have..... Not only at the age of two and definitely not the age of five or six.	FGD-8	PM007
Life Impact	Delivery of care	Parental preparedness to discharge<br>Staff education and training<br>FCC	I would say that with a healthcare professional, we look at how much aaaa in terms of preparedness with information that they have been provided. I don't know how to give it in what term. But the level of education on family-centred care program. Ummm You know them like that. Those are their knowledge, their level of knowledge on families and care program should should be be tested as well and also something, we should be able to measure what the barriers are in the delivering what they've been taught.	FGD-8	PM006
Life Impact	Delivery of care	Communication between staff and parents<br>Parental role	Mindset arguments. Tell me about it again mindset And I agree. So yeah, and communication and staff like that. So, with a heck of a top of my head, come up with. There's a lot I could come up with. Then if you move to the parents, for example. Now, again. I know you were trying to ask that you know um baby you know how do we position the level of importance of these stakeholders? Again, I'm hoping that we can present all partners as key .. as important importance.	FGD-8	

			Equal as much as possible. Because the moment we put this say that this parent is like this, then we still have the gap.		

Core Area	Outcome Domain	Outcome	Illustrative Quotes	File	Participant Code
Life impact	Role functioning	Parental presence	I agree that there is the importance is to have the parents not only as sometimes the wording is used as allowing parents to be in the NICU, ummm It's really a welcoming policy, what needs to be implemented. To have not visiting hours so that parents have the opportunity to be there for 24 hours whenever they are also half the time, especially when fathers or partners go back to work so that there is not the limitation of having access to your baby.	FGD-9	PM008
Physiological/Clinical	Physiological health	Neurodevelopment	the infant and then the family centred developmental approach because it is important to have a proper brain development, proper development later in life and also ensure that when discharge is coming.	FGD-9	PM008
Life impact	Role functioning	Parental confidence<br>Parental preparedness discharge<br>Parental involvement in infant care<br>Parental presence	That the parents are self-confident and prepared for going home because this baby is going home not with the nurse and with the doctors, it's going home with the parents. And so therefore it needs to have ummm a complex approach. So the infant, the family, the development of the brain and also development of the bonding of the the family and the baby. .....And at the end, planning really a proper discharge management and the integrated care means also from the wording to integrate the parents really in this approach. For me as a patient, it's important to that this really comes to a, yeah, it's a normal standard to integrate the parents in the care of the baby. They are the main caregivers or should be the main caregivers. Of course, not for complex medical treatment. But for the care of their baby because they are the parents of the baby. And parents are parents and no visitors, no people who are standing next to the nurse, they should actively be involved.		
Life impact	Role functioning	KMC<br>SSC<br>Parent -infant interaction	1970s when we when when aaa different people came up that parents should be involved, that kangaroo care or skin to skin care is is important and very helpful at this time it was very helpful not seen as it is today as a scientific treatment, really, and such an improvement what skin to skin care makes and also the voice of the family. Aaa Especially of the mother and the smell and everything.	FGD-9	PM008

Life Impact	Delivery of care	Bonding<br>Parental confidence after discharge	We had not infant family centred care, and we had not integrated the care we had visiting hours three hours a day with one chair for the whole unit. And at this time, there was no focus at all. Radio was running 24/7 to entertain the the team in the NICU because it was there was no quiet it was not quiet. It was noisy loud and even Lucas afterwards six months when he was at home. He needed radio 24/7 to be able to sleep. And so, this is something what I think it is important to really think not only on the integration, on the family, on the infant. It is important to have a healthy baby afterwards and therefore it's important from the very beginning to integrate in the system. When the whole infant family-centred system the development of the care of the outcome of the child and also of the bonding.	FGD-9	PM008
Life impact	Role functioning	Parental role	when like in our case we had to learn the first year when we come home to be a parent. We were we were overwhelmed with now I'm I'm the parent a day before I was just a visitor. But the next day I was the parent and was fully responsible for Lucas. And the day before, I was even not allowed to feed my baby. And so, this is something this is also part for me of development. Development to be self-confident, to go home.	FGD-9	PM008
Physiological/Clinica l	Physiological health	Infant pain<br>Infant stress	And also, what does it mean to reduce stress for babies, pain, noise? Any kind of other aspects to ensure a healthy development.	FGD-9	PM008
Life Impact	Delivery of care	Family unity	when I have my preterm twins 27 years ago In Germany, for example, there were still visiting ours and umm there were not chairs for every parent for example and there was only um permission for the parents themselves, not for siblings, not for grandparents and we were lucky the grandparents could even see and visit our child.	FGD-9	PM008
Life impact	Role functioning	Cultural effect on parental involvement in NICU	they need to stay at home because that it comes from the Yeah, from the very old times, I would say that a woman, when she gives birth, she's still bleeding for between four and six weeks. It's also in some countries in Africa or in Muslim countries that the woman is not allowed to come because she's not allowed to leave the house because she's still bleeding.	FGD-9	PM008

Life Impact	Delivery of care	Organisational factors	Then you would keep them together. But in China. It's not existing at all, and they have visiting hours, and they also have the regulation to pay the bill. That means you have to pay every week your bill and if not, the treatment cannot continue. That's reality. Yeah.	FGD-9	PM008
Life Impact	Delivery of care	Nosocomial infection	Mm-hmm. Yeah, the problem is in all countries that you have the publications, for example, also for infection. There's there is evidence. that the infection is not coming from the parents. The infection comes from, if it comes from healthcare professionals. To go from baby to baby and have not enough time and resources maybe to wash your hands or disinfect your hands. That is evidence.	FGD-9	PM008
Life Impact	Delivery of care	Accommodation Travel	what we also have to take into account, and that is also important to understand it from a global perspective, for example, in Africa. When you go to a hospital, and you need four hours to go there. And at home you have further five children Who is taking care of them. And so in our first world environment and where and it would not say everything is organized but many things are organized or most of the things or there's financial resources or their families or any kind of other support is available somehow. There is nothing available. That means they cannot even afford. I have seen this in Kosovo, for example, when I was also in Bulgaria. In Romania, they cannot even afford the bus transport for themselves to visit their baby that go two or three hours one way, have two or three children at home. How should they organize this? Financially, I can remember I was four and a half months in the hospital. We had to cover everything ourselves. We had to finance the barrier of our daughter. We were financially ruined at this time. So, we needed a credit to really finance the time in these four and a half months and it was a disaster. And I mean, we live in Munich in a rich environment. And that's at the end something what you just have to copy in an environment where one way needs four hours and you have to pay the bus ticket every day And you have four children at home. It is really important to see The whole complexity and this is why it is important to have not only financial support or resources for the family to go to the hospital.	FGD-9	PM008

Life Impact	Delivery of care	Family unity	How.. What is available, what can be available to support the family at home? To manage this because a child is not one week in the hospital a NICU baby is there for several months. And this is something what we just when we see it on a global perspective, just to have this in mind and also the centralization like in Canada or in Sweden, there are just a few hospitals and they have distances of four hours driving or in Canada, four hours flying to come to the next hospital. And so what does this mean for a family and how can we ensure a family integrated centred care approach When the rest of the family four and a half months is lost	FGD-9	PM008
Life Impact	Delivery of care	Government support	That is something what needs to be taken into account when we talk about this approach itself that families need to have places to stay, they have to have food they have to have support at home. And what do we do even when it is a single mom? So, there are a lot of things around when we talk about infant and family centred care and we have to think about the whole family and not only in the time in the NICU, what kind of support this family needs to be able to be with their baby. And this is from a financial perspective for a government for a society, something we have to think about.	FGD-9	PM008
Life Impact	Role functioning	Parental presence	But you said about the UK perspective, even in the UK, to be honest um as a developed country and we talk a lot about family integrated care actually when you really dig deep and ask a unit, do you deliver family integrated care then the the response is always yes. But actually, when you then ask, oh, do all your parents then stay 24 hours do you have the facilities for them to stay actually realistically, aaa the real answer to that is no, they don't because there isn't the environment. There isn't we know some units even in the UK need renovation. They need, you know, I guess we need some new hospitals, we need new units and so there isn't practical terms there just isn't the space for all the parents to be there um And that's just due to basic sort of practicalities, the environment itself, the neonate unit itself.	FGD-9	PN003

Life Impact	Delivery of care	NICU design and polies	And I've even been I've worked previously hospital in East London where they are brand new hospital millions of money went into it and the neonatal unit was renovated. Well, not renovated it was part of a new hospital and they um I think there were two parent rooms in a 30 bedded unit for parents to stay and that's that is that is in recent times. So actually, whereas the other the other sort of side of that is that I know in Liverpool they're now developing, I don't know how far they've got with it they are building a new unit which with a very different philosophy where they I mean I haven't been to visit there but I've heard that it's It's you know it's designed to facilitate family integrated care. But that has come in with the planning of the new unit. But, you know, we have to accept again that that doesn't necessarily always happen even though there might be a philosophy of family integrated care but actually in practical terms is it actually really possible? That's not being negative	FGD-9	PN003
Life Impact	Delivery of care	Staff attitudes towards FCC	because I think as long as staff understand the importance of it, they can do you know they can involve and integrate the parents you know ward rounds hopefully again that's very variable um but you know in those in the decision making even if they don't have the environment to be able to do it.	FGD-9	PN003
Life impact	Delivery of care	Parental satisfaction	I think it has to be parent satisfaction, doesn't it? Whatever measure you decide to developed for that but that say satisfaction I suppose yeah um what is their experience and has it improved or somehow measuring umm parents views parents opinions in terms of ummmm I suppose, yeah, I suppose it's experience isn't it what is what is their experience and how do they view that their Because otherwise… because they're the ones that we're that are the centre of because obviously we can't ask the baby and we don't want I guess you can you can ask the the views of staff but ultimately has to come back to the parents doesn't it in terms of yeah a parent satisfaction measure or a however you again whatever term you use for that…improvements in that. I don't know. I mean, I mean, there must be measures that are around already in terms of surveys and tools so ummm I think it's an area that's been looked at in research, hasn't it, before? In terms of looking at patient slash parent satisfaction about the care that they've received.	FGD-9	PN003

Life Impact	Emotional function/wellbein g	Quality of life<br>Parental stress	I think what also is important is measuring the quality of life for the parents during the NICU and also afterwards because you can measure very very nicely the stress level of parents in the NICU and afterwards depending on if family-centred care was implemented or not for sure you will find outcomes.	FGD-9	PM008
Life impact	Delivery of care	Infection<br>Staff educate and training<br>Cost savings	I would also because hard data are always the reduction of infection to an integrated care umm is something what you can because this is always the argument like you said before in China to have have ummmmm If you have implemented, do you have less actions? Of course, you need then to to to educate and train the parents before about hand washing and other hygiene aspects. This could be aaa beneficial.	FGD-9	PM008
		Exclusively breastfeeding at discharge	Also, the breastfeeding rates, umm I would measure to see if the breastfeeding or fully breastfeed the babies are going home when you really or have you more moms who are able to breastfeed or to continue breastfeeding. Would be something which would be a hard argument.	FGD-9	PM008
Physiological/Clinica l	Physiological health	Heart rate<br>SSC	Also, the health outcome is something what could be measured to, for example, heart rate ummm and because it's that this can be helpful because family-centred care approaches also integrates normally skin to skin contact as long as possible.	FGD-9	PM008
Resource use	Hospital environment and resource use	LOS	And what I also would measure, and this is an argument for for money is the length of stay because length of stay is something what Karolinska has measured and one family, for example, one day cost 1,600 euros. And when you aa reduce the length stay only for four days, what Karolinska showed and also another group has shown six or seven days. Just…And when you then see all the children you have per year that would be something which is an argumentation for the health for the hospital administration, so the directors And of course, for the society, because money is one of the biggest arguments.	FGD-9	PM008
Life Impact	Emotional function/wellbein g	Quality of life<br>Stress level<br>Post traumatic disorder	you can have emotion is, of course, also, but when you really measure emotion, I would recommend to have quality of life because then we'll measure the stress level. And the stress level means at the end aaa potential health risk factors for parents, aaa especially for the moms.	FGD-9	PM008

Resource use	Hospital environment and resource use	Policy<br>Cost savings	And this is something what also could in the end argue again for the society, more health costs later in life because the parent potentially has higher risk of post-traumatic stress syndrome about having longer ummmm a sick leave, for example, and this is because I'm always policy level and you have to convince people of course with arguments and one argument is money And money is always very helpful to get things implemented. And this is what we are also doing when we do our job.	FGD-9	PM008
Life Impact	Delivery of care	Staff workload	To argue not only about emotions, we are arguing about what is the benefit for the hospital maybe you have a lack of nurses then you have ummm when you have parents who are there, they can support with feeding they can observe the baby and see earlier any kind of health issues. Ummmm and so, there's often an argumentation to to the hot liner we always say to be on implementing such a system just some thoughts.	FGD-9	PM008
Physiological/Clinical	Physiological health	Weight gain<br>Growth	I mean I think I mean when you look at the work from um I can't remember is it lee isn't it who the the research on family integrated care they did they measured things like growth and weight gain	FGD-9	PN003
Physiological/Clinical	Physiological health	Parent-infant interaction Infant stress<br>Neurodevelopment<br>Infant pain	Pierre Kuhn from Strasbourg, he has also measured the stress level with an MRI. Oh, right. Mri? Is it MRI? And to see that in the end, the children who have um especially the mother always with her and hear the voice and smell the mother that they have a lower stress level. Hmm. And so you can... if you have enough money for sure also measure later on a better um well this is a hypothesis but um a better brain development because when you have continuously four and a half months stress then it is not good for your brain development. But what he measured and what he found out is that children who have the parents, especially the mom always with them because of being skin to skin care having the mom helping for painful treatment for example that these children, then this is what they could measure, have a lower stress level. And even when there were painful procedures and the mom was there and they sucked a little bit on breast milk and they They integrated in the end this concept These children even felt less les pain because of this kind of treatment they have provided. And compared to the others.	FGD-9	PM008

Physiological/Clinical	Physiological health	Parental involvement during medical procedures<br>Breastmilk<br>Heart rate<br>Stress	So, for example, do extubating on skin-to-skin position or do painful procedures when the mom is holding the baby and sucking on a stick or something on breast milk. This is such a complexity. It means not only having the mom next to the incubator. That's not the concept. The concept is really this active involvement, and this is so complex that it makes it difficult in the end to also measure things because if you measure, for example, the stress level that's one thing. And if you measure in the end the amount of breast milk this baby gets or their heart rate to reduce the heart rate to be stabilized to have an earlier discharge. And in the end, at home it's mainly better than in the hospital. So, these are so many things of of the aspects and the challenge is often that you have these niches.	FGD-9	PM008
Life Impact	Emotional function/wellbeing	Father depression<br>Grandparents' emotional well-being	I just want to add because we were also I talked a lot about the mother because my husband had deep depressions and also health challenges afterwards. I want to stress that when we talk about, of course, family-centred care, that we don't think that this is only the mom. It is really the whole family and to not forget the fathers or partners and also other family members like siblings, grandparents can remember that my parents suffered enormously really on on the the situation.	FGD-9	PM008
Life Impact	Emotional function/wellbeing	Family support	And this is something what means family as well, not focusing only on the mom thinking on the partner father of the baby, the siblings, other family members, or even maybe close friends. Because these are the people who after the hospitals will hmmm suffer maybe or will be supportive for the little family and so this is why umm when we talk about this, it really means a whole family.	FGD-9	PM008

Life Impact	Emotional function/wellbeing	Family emotional support	Yeah, I think that's a really important point, actually, because when you talk about family maybe you know that needs to come into the early question around definition. Remembering it you know parents obviously very very important, but the family And I think siblings definitely But grandparents are you know absolutely often absolutely essential part of the support network for parents themselves aren't they? I mean, I I one of my key speakers for my neonatal module is a grandmother who comes to speak to my students now. Who supported ….her granddaughter was born around 24 weeks and so was there all the time with her daughter at the time who at the time didn't have a partner so it was mum. But it was interesting her story you know is that she sort of had to fight in a way fight. You know as in be quite upfront about the need to be there in the neonatal unit and she didn't, you know, again, it was a bit of a fight for her because she ummm was told that, you know, there were visiting times for her which prevented her from being there with her daughter and supporting her. So, she did have to fight a bit to be able to be there which is a shame because she's an absolutely central part of the family. So, her experience really I think reitates to me the family is much, much wider isn't it?	FGD-9	PN003
Life impact	Delivery of care	Government support	And it should not be underestimated the long-term health consequences of such a situation so the trauma which has it for the whole family. And so, this is why it is important that the parents are actively involved, but also that they get support like psychological support or social support to ensure that afterwards they have not health issues later and that can be the family. And this is why I said about the quality of life, it is important to measure to see and because this is the argumentation of getting support. If you do not have data, if you have numbers you do not get financial support from a government on health insurance or whatever. So you need this data….. So that it's not only, oh, yes, this is traumatic, this is an emotional that's it. If you measure it, if you show the consequences even later in life for the family on a health aspect, on financial burden for the society.	FGD-9	PN003
